# Sas20 is a highly flexible starch-binding protein in the *Ruminococcus bromii* cell-surface amylosome

**DOI:** 10.1016/j.jbc.2022.101896

**Published:** 2022-04-01

**Authors:** Filipe M. Cerqueira, Amanda L. Photenhauer, Heidi L. Doden, Aric N. Brown, Ahmed M. Abdel-Hamid, Sarah Moraïs, Edward A. Bayer, Zdzislaw Wawrzak, Isaac Cann, Jason M. Ridlon, Jesse B. Hopkins, Nicole M. Koropatkin

**Affiliations:** 1 Department of Microbiology and Immunology, University of Michigan Medical School, Ann Arbor, Michigan, USA; 2 Department of Animal Sciences, University of Illinois at Urbana-Champaign, Illinois, USA; 3 Carl R. Woese Institute for Genomic Biology (Microbiome Metabolic Engineering Theme), University of Illinois at Urbana-Champaign, Illinois, USA; 4 Faculty of Natural Sciences, Life Sciences, Ben-Gurion University of the Negev, Beer-Sheva, Israel; 5 Department of Biomolecular Sciences, The Weizmann Institute of Science, Rehovot, Israel; 6 Northwestern University, Synchrotron Research Center, Life Science Collaborative Access Team, Lemont, Illinois, USA; 7 Biophysics Collaborative Access Team, Illinois Institute of Technology, Advanced Photon Source, Argonne National Laboratory, Lemont, Illinois, USA

**Keywords:** carbohydrate-binding module, resistant starch, amylosome, *Ruminococcus bromii*, B-PNP-maltoheptaose, benzylidene-blocked para-nitrophenyl maltoheptaoside, CBM, carbohydrate-binding module, CBM-Coh, CBM-fused cohesin, GH13, glycoside hydrolase family 13, ITC, isothermal titration calorimetry, MS, mass spectrometry, MW, molecular weight, NIGMS, National Institute of General Medical Sciences, PDB, Protein Data Bank, PNP, para-nitrophenyl, PSM, peptide-spectral match, RS, resistant starch, SAD, single-wavelength anomalous dispersion, Sas20, starch adherence system protein 20, Sas20d1, domain 1 of Sas20, Sas20d2, domain 2 of Sas20, Sas20d1tr, truncated version of Sas20d1, SAXS, small-angle X-ray scattering, Sus, starch utilization system

## Abstract

*Ruminococcus bromii* is a keystone species in the human gut that has the rare ability to degrade dietary resistant starch (RS). This bacterium secretes a suite of starch-active proteins that work together within larger complexes called amylosomes that allow *R. bromii* to bind and degrade RS. Starch adherence system protein 20 (Sas20) is one of the more abundant proteins assembled within amylosomes, but little could be predicted about its molecular features based on amino acid sequence. Here, we performed a structure–function analysis of Sas20 and determined that it features two discrete starch-binding domains separated by a flexible linker. We show that Sas20 domain 1 contains an N-terminal β-sandwich followed by a cluster of α-helices, and the nonreducing end of maltooligosaccharides can be captured between these structural features. Furthermore, the crystal structure of a close homolog of Sas20 domain 2 revealed a unique bilobed starch-binding groove that targets the helical α1,4-linked glycan chains found in amorphous regions of amylopectin and crystalline regions of amylose. Affinity PAGE and isothermal titration calorimetry demonstrated that both domains bind maltoheptaose and soluble starch with relatively high affinity (*K*_*d*_ ≤ 20 μM) but exhibit limited or no binding to cyclodextrins. Finally, small-angle X-ray scattering analysis of the individual and combined domains support that these structures are highly flexible, which may allow the protein to adopt conformations that enhance its starch-targeting efficiency. Taken together, we conclude that Sas20 binds distinct features within the starch granule, facilitating the ability of *R. bromii* to hydrolyze dietary RS.

The human gut microbiota, the dense and heterogeneous consortium of bacteria that reside in the intestinal tract, has a profound influence on host health and disease (1, 2). Dietary fiber feeds this community and dictates the bacterial fermentation profile of short-chain fatty acids that mediate several host responses (3). Resistant starch (RS) is one such dietary fiber that tends to shift our gut bacterial community to one that promotes health (4). While much of the processed starch in our diet is degraded by host or bacterial enzymes in the small intestine, a fraction of dietary starch resists enzymatic degradation and transits the large intestine. In the distal part of the gut, few specialized members of the microbiota can utilize RS (5, 6). There are different types of RS classified according to the mechanism by which they are resistant to host intestinal enzymatic processing (7). While not all RS has similar effects on our microbiome (8), RS consumption tends to increase colonic butyrate, a microbially derived short-chain fatty acid that strengthens the gut barrier and has anti-inflammatory and anti-tumorigenic properties (9, 10, 11, 12).

*Ruminococcus bromii* is a primary degrader of RS and is considered a keystone species as it crossfeeds starch breakdown products to other bacteria in the gut (5). *R. bromii* organizes its starch-binding and starch-degrading proteins into one or more extracellular complexes called amylosomes (13, 14). Akin to multiprotein cellulosome complexes synthesized by Gram-positive organisms for the degradation of cellulose, amylosomes are assembled *via* calcium-dependent protein–protein interactions (15, 16). Like cellulosomes, amylosomes are built around a structural protein called a scaffoldin that possesses one or more cohesin modules. These cohesin modules bind to dockerin modules on secreted starch-targeting enzymes and binding proteins, creating a complex that hydrolyzes starch (6, 13, 14). Biochemical studies on the recombinantly expressed cohesin and dockerin modules have revealed that there is a number of potential interactions among putative amylosome proteins (13, 14). This suggests that there may be more than one type of amylosome synthesized, perhaps allowing the cell to respond to different environmental conditions, as has been observed for cellulosomes (17, 18).

A key feature of enzymes that degrade insoluble fibers like RS is the presence of carbohydrate-binding modules (CBMs) (19). CBMs are auxiliary modules of ∼100 amino acids that bind to substrate and thus enhance enzymatic efficiency (20, 21). CBMs are classified by amino acid sequence, and there are currently 15 CBM families that target starch (6, 22). While the precise molecular recognition varies, starch CBMs generally have a curved aromatic platform that complements the natural helical turn of the α1,4 glycosidic bond (19). This molecular feature is also observed within the proteins of the starch utilization system (Sus) from the Gram-negative human gut bacterium *Bacteroides thetaiotaomicron*. The Sus features three cell surface–exposed starch-binding lipoproteins (SusDEF) and a single glycoside hydrolase 13 enzyme (SusG) that targets α-glucans such that starch binding and hydrolysis are split across the four proteins (23). Numerous examples of Sus-like complexes, comprised of glycan-binding proteins and enzymes that target many other carbohydrates, have been studied in detail in several *Bacteroides* species (24, 25, 26, 27). Other examples of bacterial complexes that include both noncatalytic carbohydrate-binding proteins and enzymes include cellulosomes from Gram-positive bacteria, in which both enzymes and carbohydrate-binding proteins dock to the scaffoldin, which may also feature carbohydrate-binding domains for docking to cellulose (28, 29).

Bioinformatic analysis of the *R. bromii* genome identified five scaffoldin proteins with cohesin domains (Sca1–5) and 27 proteins with dockerin domains (13, 14). Only five of these dockerin-containing proteins have predicted glycoside hydrolase family 13 (GH13) catalytic modules that are specific for α-glucan degradation. This leaves 22 proteins, originally called “Doc” proteins 1 to 22, that may be incorporated into the amylosome. Many of these proteins likely bind starch, creating a system of starch-adhering proteins that help tether the bacterium to RS granules. Here, we extend our previous work on the amylosome by characterizing one such dockerin-containing protein that assembles into this complex that we have named Sas20 for starch adherence system protein 20. Using a combination of X-ray crystallography, small-angle X-ray scattering (SAXS), and isothermal titration calorimetry (ITC), we demonstrate that Sas20 is a highly flexible starch-binding protein comprised of two domains with different starch-binding features. These data extend our molecular understanding of how a keystone human gut bacterium targets RS in the gut.

## Results

### Sas20 is a component of cell-surface amylosomes

Previous work using the cohesin domain from Amy4, a cell-surface amylosome protein, as a probe to capture amylosome proteins from fractionated *R. bromii* cells identified Sas20 (previously named Doc20) as one of the more abundant proteins (13). In the same study, Sas20 was also identified as one of the major proteins found in the cell pellet and cell culture supernatant of *R. bromii* cells grown on soluble starch. Following on these results, we sought to identify proteins that make up the cell-surface amylosome network by leveraging the calcium-dependent nature of cohesin–dockerin assembly (30, 31). *R. bromii* cells were grown in either galactose or autoclaved potato amylopectin to early stationary phase, washed with PBS, then incubated in PBS with or without 10 mM EDTA to disrupt cohesin–dockerin interactions (see the Experimental procedures section) (14). Proteomic analysis of the washed cells revealed many peptide-spectral matches (PSMs) to predicted amylosome proteins, with an enrichment of these proteins in the EDTA-treated sample (Table 1, all data in Table S1). Amy4, an amylase with both a cohesin and dockerin module, had the highest number of PSMs in the EDTA samples. Interestingly, Amy1 and Amy2, secreted amylases that lack predicted cohesin or dockerin modules, were also higher in the EDTA wash. This may suggest that not all amylosome proteins interact *via* cohesin–dockerin interactions. Sca2 and Sca5, scaffoldin proteins that encode sortase recognition sequences, represented a negligible amount of the peptide repertoire in the PBS- or EDTA-wash conditions. Sas20 was also a protein for which there were more PSM assignments from the EDTA wash compared with the PBS wash in cells grown in either galactose or potato amylopectin. Intrigued by the recurring presence of Sas20 as an amylosome component across studies and its low sequence homology to characterized proteins, we performed a structure–function study of Sas20 to determine its role in the *R. bromii* amylosome.

**Table 1:** Highest abundant proteins from EDTA elution

Locus tag	Name	No. of amino acids	PBS gal PSM	AVG EDTA gal PSM	PBS amylo PSM	AVG EDTA amylo PSM	Domain architecture
L2-63_00682	Amy4	1356	19	107 ± 11.3	29	210.5 ± 2.1	SP GH13 CBM26 CBM26 Coh Doc
L2-63_00496	Amy2	751	17	76 + 9.9	29	128.5 ± 16.3	SP CBM26 GH13
L2-63_00433	Amy1	804	28	76.5 + 4.9	31	117.5 ± 7.8	SP CBM26 GH13
L2-63_01094	Amy10	1233	5	77 ± 14.1	2	115.5 ± 2.1	SP CBM48 GH13 MucBP MucBP CBM26 MucBP Doc CBM26
L2-63_01654	Amy16	876	11	68.5 ± 9.2	18	89.5 ± 4.9	SP GH13 CBM26 Doc CBM26
L2-63_00434	Doc22	548	12	16.5 ± 2.1	6	53 ± 1.4	SP CBM26 CBM26 DUF Doc
L2-63_00125	Sas20	630	6	40.5 ± 4.9	15	49 ± 1.4	SP Sas20d1 Sas20d2 Doc
L2-63_01357	Amy12	1059	0	23 ± 0.0	1	32.5 ± 3.5	SP CBM48 GH13 MucBP Doc MucBP CBM26
L2-63_02041	Amy9	1056	8	14 ± 1.4	25	30 ± 1.4	SP GH13 CBM26 Doc
L2-63_01861	Doc8	245	19	17 ± 0.0	14	22.5 ± 2.1	SP DUF Doc
L2-63_00436	Doc14	550	0	22.5 ± 0.7	0	21 ± 1.4	SP PEP A-S Doc
L2-63_00285	Doc1	549	2	16.5 ± 2.1	1	20.5 ± 2.1	SP LRR LRR Doc
L2-63_01443	Doc6	734	2	11.5 ± 3.5	1	17.5 ± 0.7	SP DUF Doc
L2-63_00287	Doc2	471	2	13.5 ± 2.1	2	15 ± 1.4	SP LRR Doc
L2-63_00780	Amy5	551	4	4.5 ± 0.7	4	10 ± 2.8	SP GH13

Abbreviations: Amylo, autoclaved potato amylopectin-grown cells; A-S, NAD(P)+-dependent aldehyde dehydrogenase superfamily; AVG, average; DUF, domain of unknown function; Gal, galactose-grown cells; LRR, leucine-rich repeat; PEP, peptidase; SP, signal peptide.

Common contaminants and cytoplasmic proteins were omitted. PBS samples n = 1. EDTA samples are average of n = 2.

**Figure 1:**
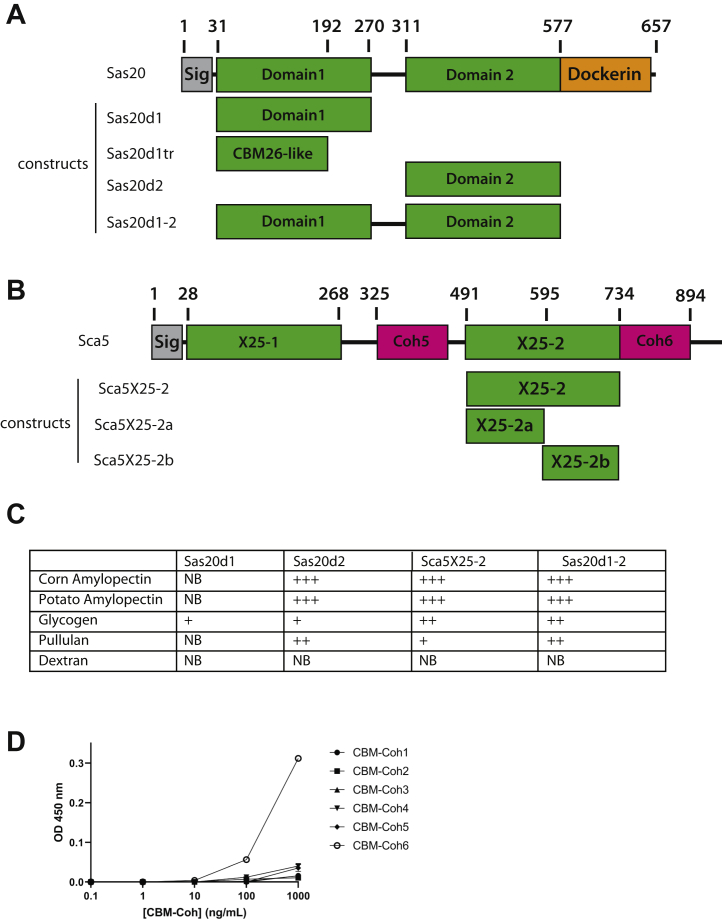
**Protein constructs and affinity PAGE results.***A*, Sas20 constructs used in this study. *B*, Sca5 constructs used in this study. *C*, summary of affinity PAGE results for select polysaccharides; gels are presented in Fig. S1. *D*, functionality of the Sas20 dockerin as measured by ELISA. A microtiter plate was coated with Xyn-Sas20. Positive interaction of the Sas20 dockerin was observed with Coh6. Error bars indicate SD from the mean of duplicate samples from one experiment. Coh6, cohesin 6; NB, no binding; Sas20, starch adherence system protein 20.

Sas20 is a protein of 657 amino acids that has an N-terminal secretion signal, two predicted globular domains, and C-terminal dockerin domain (Fig. 1*A*). Domain 1 of Sas20 (Sas20d1) has no significant sequence homology to any proteins in the Protein Data Bank (PDB) and no sequence similarity (E value <0.05) to characterized proteins. Domain 2 of Sas20 (Sas20d2) has distant homology to the X25_BaPul-like family of starch-binding domains (E value = 10^−6^) (32). A linker of 41 amino acids rich in Thr/Pro separates Sas20d1 and Sas20d2. Interestingly, Sas20d2 shares 81% sequence identity with residues 491 to 734 of Sca5, hereafter referred to as Sca5X25-2 as it is the second X25-containing domain in the sequence. Therefore, we included this domain in our analysis (Fig. 1*B*). Sca5 is an 894 amino acid scaffoldin protein that also has an N-terminal secretion signal, two X25 modules, two cohesin modules, and a C-terminal sortase sequence (14).

We created the construct Sas20d1-2 that lacks the dockerin module and secretion signal as well as the individual domains Sas20d1, Sas20d2, and Sca5X25-2 to determine their potential for starch binding *via* affinity PAGE (Figs. 1*C* and S1) (33, 34). In this method, protein binding is qualitatively assessed by a decrease in mobility through nondenaturing gel upon interaction with polysaccharide. For this analysis, we tested the soluble polysaccharides amylopectin, glycogen, pullulan, and dextran. Amylopectin is one of the two polysaccharides within starch granules and contains both α1,4 and α1,6 linkages, whereas glycogen, found in animals and bacteria, has a higher proportion of α1,6 branches (35, 36). Pullulan is found in fungal cell walls and is a linear polysaccharide of maltotriose linked by α1,6 linkages (37, 38). Sas20d2, Sca5X25-2, and Sas20d1-2 bind to corn and potato amylopectin with relatively high affinity as suggested by their retention at the top of the gels but demonstrated more moderate binding to glycogen and pullulan (Fig. 1*C*). These data suggest that Sas20d2 and Sca5X25-2 accommodate α1,6 linkages but that binding is likely driven by binding to α1,4 glucan regions. While Sas20d1 only showed modest affinity to glycogen in this assay, we could quantify its binding to amylopectin *via* ITC (described later). We speculate that our inability to observe binding by Sas20d1 in this assay may be due to incompatibility of the protein with the electrophoresis conditions, as some aggregation may occur in the nondenaturing gel. None of the constructs bound dextran, an α1,6-linked glucan, underscoring the specificity of the Sas20 and Sca5 domains for α1,4-linked starch components.

To determine how Sas20 is assembled into the amylosome system, a standard affinity-based ELISA procedure was performed by using a fusion construct including the dockerin module from Sas20 (39). We tested binding to the six known cohesin modules in the *R. bromii* genome (CBM-fused cohesin [CBM-Coh]1–6) and discovered that the Sas20 dockerin module interacts specifically with CBM-Coh6, the second cohesin of the anchoring scaffoldin Sca5 (Fig. 1*D*). These data support the results of our proteomic experiments and suggest that Sas20 is a component of the cell-surface amylosome *via* its interaction with Sca5 and likely aids in the docking of *R. bromii* to starch granules.

### Sas20d1 structure

**Figure 2:**
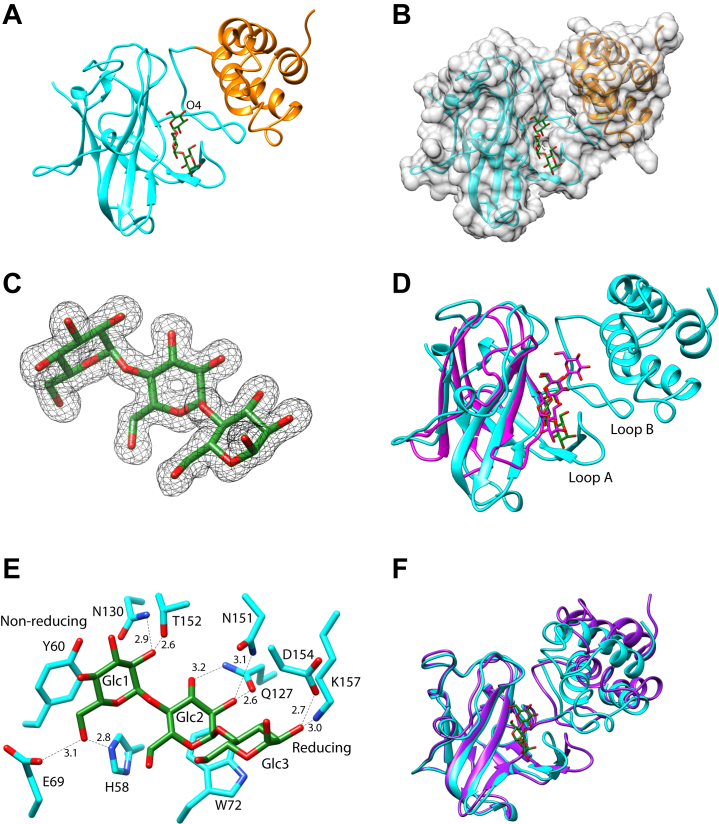
**Sas20d1 structure.***A*, cartoon of Sas20d1 with maltotriose (*green*) with the β-sandwich (residues 34–190) in *cyan* and α-helical bundle (residues 191–268) in *orange*. *B*, surface rendering of Sas20d1 structure demonstrating capture of maltotriose between the β-sandwich and helices. *C*, omit map of maltotriose, σ = 3.0. *D*, structural alignment of Sas20d1 with maltotriose (*cyan*) and CBM26 (residues 279–387) with maltotetraose from Amy13k (ErCBM26; PDB: 6B15, *magenta*). Residues 146–161 make up loop A; residues 169–189 make up loop B of Sas20d1. *E*, close-up view of maltotriose-binding site in Sas20d1 as colored in *A*. Hydrogen bonds are depicted as *black dashed lines* and with distances in angstroms. *F*, overlay of the Sas20d1 native (*purple*) and maltotriose-bound (*cyan*) structures. CBM26, carbohydrate-binding module family 26; PDB, Protein Data Bank; Sas20d1, domain 1 of Sas20.

We solved the crystal structure of Sas20d1 *via* sulfur single-wavelength anomalous dispersion (SAD) phasing (2.1 Å, *R*_w_ = 17.7%, *R*_f_ = 21.4%) and then used this as a model to determine the structure with maltotriose (1.5 Å, *R*_w_ = 17.5%, *R*_f_ = 19.7%; Table 2). Sas20d1 has a canonical β-sandwich CBM fold at the N terminus with a bundle of three α-helices at the C terminus, with maltotriose accommodated between these features (Fig. 2, *A*–*C*). The N-terminal β-sandwich most closely resembles a CBM26 module, which can be found adjacent to catalytic domains on α-amylases and typically binds maltoheptaose and β-cyclodextrin (19, 40, 41, 42). A search on the DALI server showed that CBM26 from the *Eubacterium rectale* α-amylase Amy13K (ErCBM26) had the highest structural homology to Sas20d1 and aligns with an RMSD of ∼2.3 Å over 85 Cα atoms (Fig. 2*D*) (43, 44). While ErCBM26 and Sas20d1 share a conserved β-sandwich fold, two long loops formed by residues 146 to 161 (loop A) and 169 to 189 (loop B) protrude from Sas20d1 and are not found in ErCBM26. These two loops are near the maltooligosaccharide-binding interface, and residues of loop A provide a hydrogen-bonding network for the O2 and O3 hydroxyls of the ligand (Fig. 2, *D* and *E*). Maltotriose is primarily bound at the β-sandwich surface of Sas20d1 *via* the aromatic platform created by Y60 and W72. The nonreducing end O4 is directed toward the small solvent-filled cavity between the β-sandwich and the α-helical bundle and does not directly interact with the protein (Fig. 2, *B* and *E*). The O2 of Glc1 is positioned 2.6 and 2.9 Å away from the side chains of T152 and N130, respectively. Q127 makes hydrogen bonds with Glc2 O2 and O3, whereas the side chain of N151 is located 3.1 Å from Glc2 O2. At the reducing end, Glc3 has little direct interaction with the protein, with O2 positioned 3.0 and 2.7 Å away from the side chains of K157 and D154, respectively. While we later show that Sas20d1 binds maltoheptaose with enhanced affinity over maltotriose, our attempts at cocrystallization with maltoheptaose failed to demonstrate additional density at the nonreducing end, and only disordered density for an extra glucose at the reducing end, likely because of lack of productive interaction with the protein (data not shown). The φ (O5-C1-O4’-C4′) and Ψ (C1-O4’-C4’-C5′) angles of maltotriose in our structure (φ = 102.4°, Ψ = −137.3°; φ = 103.8°, Ψ = −137.9°) are more obtuse than those found in double-helical amylose (φ = 91.8°, Ψ = −153.2°; φ = 85.7°, Ψ = −145.3°; φ = 91.8°, Ψ = −151.3°) (45). Therefore, we think this domain targets more amorphous and less helical regions of starch at the nonreducing end of the α-glucan chain.

**Table 2:** X-ray data collection and refinement statistics

Parameter	Sas20 native	Sas20 maltotriose	Sca5X25-2 maltotriose
PDB accession	7RAW	7RFT	7RPY
Wavelength (Å)	0.979	0.979	0.979
Resolution range (Å)	41.13–2.10 (2.15–2.10)	30.00–1.53 (1.56–1.53)	39.27–1.67 (1.73–1.67)
Space group	I 21 3	C 1 2 1	P 32 2 1
Unit cell (Å)	*a* = *b* = *c* = 130.0	*a* = 121.8, *b* = *c* = 64.7 β = 102.8	*a* = *b* = 100.8, *c* = 87.9
Total reflections	319,452 (13,663)	339,801 (14,796)	556,138 (53,864)
Unique reflections	21,541 (1051)	74,182 (3699)	60,154 (5957)
Multiplicity	14.8 (13.0)	4.6 (4.0)	9.2 (9.0)
Completeness (%)	100.0 (100.0)	100.0 (99.9)	100.0 (100.00)
Mean *I*/sigma(*I*)	40.5 (1.2)	32.5 (1.0)	17.1 (1.3)
R-merge	0.047 (2.31)	0.047 (1.44)	0.074 (1.77)
R-meas	0.074 (2.41)	0.053 (1.67)	0.078 (1.87)
R-pim	0.019 (0.67)	0.025 (0.83)	0.026 (0.62)
CC1/2 in highest resolution shell	0.43	0.36	0.48
CC∗ in highest resolution shell	0.78	0.73	0.81
Reflections used in refinement	21,522 (1388)	70,481 (5079)	60,153 (5958)
Reflections used for R-free	1995 (144)	3699 (251)	3048 (331)
R-work	0.177 (0.281)	0.175 (0.319)	0.191 (0.309)
R-free	0.214 (0.324)	0.197 (0.328)	0.203 (0.309)
Number of nonhydrogen atoms	1921	4290	2255
Macromolecules	1793	3641	1877
Ligands	41	84	74
Solvent	111	561	304
Ions	N/A	4	N/A
Protein residues	233	464	241
RMS (bonds)	0.008	0.013	0.013
RMS (angles)	1.0	1.6	1.7
Ramachandran favored (%)	97.4	99.8	97.9
Ramachandran allowed (%)	2.6	0.2	2.1
Ramachandran outliers (%)	0	0	0
Rotamer outliers (%)	0.52	0	0
Clashscore	9.33	0.82	1.58
Average *B*-factor	66.6	24.3	25.0
Macromolecules	65.5	25.4	22.6
Ligands	98.8	24.0	34.6
Solvent	56.0	36.1	36.9
Ions	N/A	21.9	N/A

Abbreviation: N/A, not applicable.

When comparing the native and maltotriose-bound Sas20d1 crystal structures, the CBM26-like fold at the N terminus is nearly identical (Fig. 2*F*). In the native structure, the α-helices at the C terminus of Sas20d1 are somewhat disordered with elevated *B*-factors compared with the rest of the structure, but in the maltotriose-bound structure, this region is well ordered (Fig. S2*A*). The Sas20d1 crystals with maltotriose (space group C2) have 45% solvent content and a tightly packed arrangement, with a crystal contact at the helical bundle. In each monomer, the helices (residues 237–257) are sandwiched between the same helical region (residues 237–257) and two β-strands (residues 58–70) of the neighboring monomer within the asymmetric unit and a loop (residues 93–104) of a symmetry-related monomer (Fig. S2*B*). This arrangement is in stark contrast to the native crystals, which were of the cubic space group I 21 3 and have ∼62% solvent. In these crystals, there are no crystal contacts in the region surrounding the helical bundle, which in part explains the elevated *B*-factors.

In the maltotriose-bound structure, the helices move toward the ligand-binding site with a maximum displacement of ∼8 Å, although no part of this bundle directly interacts with maltotriose in our structure (Fig. 2*F*). In solution, this flexibility may allow the protein to accommodate larger ligands and facilitate the capture of nonreducing ends between the β-sandwich and the helical bundle. We used CASTp (Computed Atlas of Surface Topography of proteins; http://sts.bioe.uic.edu/castp/index.html?1bxw) to determine the size and volume of the solvent-accessible pocket created between the β-sandwich and α-helical bundle in both structures (46). Not surprisingly, the pocket of the native structure has an area of ∼783 Å^2^ and volume of ∼1350 Å^3^, whereas this space constricts to ∼521 Å^2^ and a volume of ∼848 Å^3^ in the maltotriose-bound structure (Fig. S2*C*).

### Sas20d2 homolog structure

**Figure 3:**
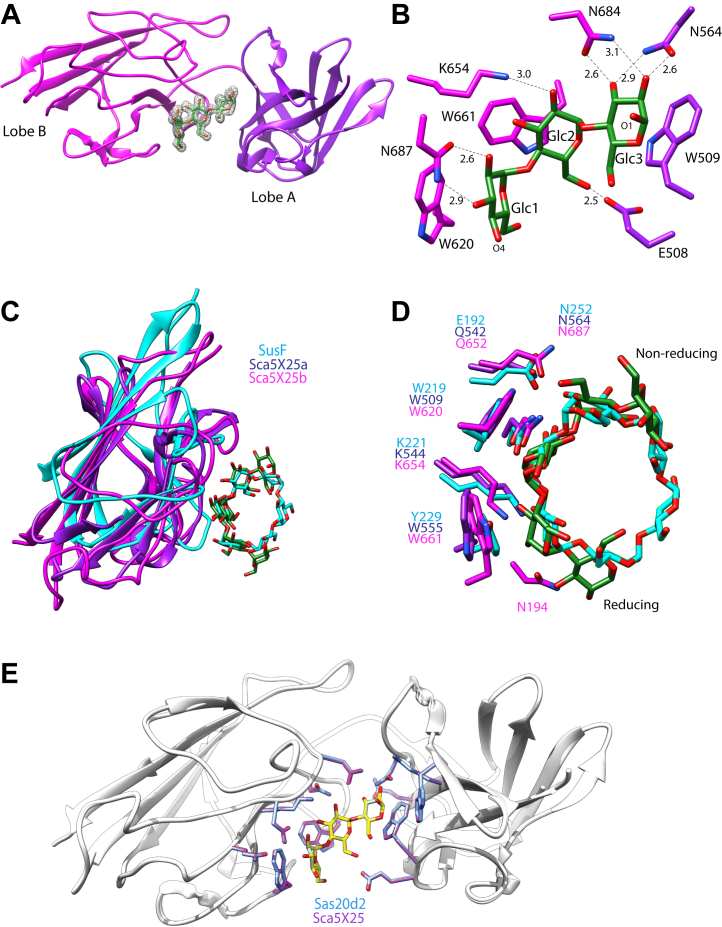
**Sca5X25-2 structure.***A*, cartoon of Sca5X25-2, with Sca5X25-2a (residues 491–595) in *purple* and Sca5X25-2b (residues 596–734) in *pink*. Omit map of maltotriose, σ = 5.0. *B*, close up of the maltotriose-binding site colored as in *A*. Hydrogen bonds are depicted as *black dashed lines*, and their distances are noted in angstroms. *C*, overlay of Sca5X25-2a (*purple*), Sca5X25-2b (*pink*), and residues 170 to 272 from α-cyclodextrin-bound SusF (PDB: 4FE9, *cyan*). *D*, close up of binding site from the overlay in *C* demonstrating the conserved starch-binding site. *E*, Phyre2 model Sas20d2 (*gray ribbon*, *blue residues*) overlaid on Sca5X25-2 (*white ribbon*, *pink and purple residues* as in *B*. The RMSD is 0.4 Å for 240 Cα. The four conserved tryptophans are numbered according to the Sas20d sequence. PDB, Protein Data Bank; Sas20d2, domain 2 of Sas20.

We could not obtain crystals of Sas20d2 but were successful in determining the structure of the Sca5X25-2 domain (residues 491–734) that is 81% identical in sequence (Figs. 1*B* and S3). The Sca5X25-2 crystal structure with maltotriose was determined by SAD phasing with selenomethionine-substituted protein (1.7 Å, *R*_w_ = 19.1%, *R*_f_ = 20.3%; Table 2). The Sca5X25-2 structure with maltotriose revealed two X25 modules in tandem, Sca5X25-2a and Sca5X25-2b (Fig. 3*A*). X25 modules fold as a β-sandwich of ∼120 amino acids and are found in tandem in the starch-binding proteins SusE and SusF from *Bacteroides thetaiotamicron* (38) and are features of some GH13 enzymes such as the *Bacillus acidopullyticus* pullulanase (24). Interestingly, both the *R. bromii* scaffoldins Sca3 and Sca5 have multiple predicted X25 modules (14). Sas20d2 and Sca5X25-2 are roughly twice the size of a single X25 domain, so we predicted two X25 modules in tandem, each with its own starch-binding site (Fig. 1*B*). However, a single maltotriose molecule was captured between these modules with amino acids from both lobes coordinating the ligand (Fig. 3, *A* and *B*). The aromatic ring of W509 in Sca5X25-2a interacts *via* van der Waals forces with the hexose ring of Glc3 at the reducing end. The O2 and O3 of Glc3 is stabilized by hydrogen bonding to the side chains of Sca5X25-2a N564 and Sca5X25-2b N684. The aromatic rings of W661 and side chain of K654 in Sca5X25-2b interact with the aglycone face and O2 of Glc2, respectively. The O6 of Glc2 is within 2.5 Å of the side chain of Sca5X25-2a E508. Glc1 interacts with W620, and its O2 and O3 coordinate with the side chain of N687. A sequence alignment between Sca5X25-2 and Sas20d2 shows that these residues within the ligand-binding cleft are conserved in the Sas20d2 sequence, suggesting that starch-binding sites in Sca5X25-2 and Sas20d2 are similar (Fig. S3). Sca5X25-1 also shares conservation of these residues suggesting that there are multiple starch-binding sites within Sca5.

Sca5X25-2a and Sca5X25-2b overlay with an RMSD of 1.0 Å over 49 Cα atoms and demonstrate a conserved binding platform; when maltotriose is included in this overlay, the ligand displays the same polarity. A search on the DALI server revealed that the Sca5X25-2a and Sas20d2-2b folds share homology with the X25 domain in the *B. thetaiotamicron* starch-binding protein SusF (PDB: 4FE9, Z-score = 7.8, RMSD = 2.5 Å; Fig. 3, *C* and *D*), including a conserved starch-binding site. W620 and W661 of Sca5X25-2a are conserved with W509 and W555 of Sca5X25-2b, although W555 was not involved in maltotriose binding in our structure. The position of W555 suggests that the binding platform shared between both lobes of Sca5X25-2 is extensive and can either accommodate longer maltooligosaccharides or allow each lobe to bind maltooligosaccharide independently. SusF has three X25 modules akin to Sca5X25-2a/b, and each recognizes maltooligosaccharides with *K*_*d*_s of ∼300 μM (47). However, for both Sca5X25-2a and Sca5X25-2b to bind individual maltooligosaccharides, there would have to be significant opening of the cleft between these lobes. The φ (O5-C1-O4’-C4′) and Ψ (C1-O4’-C4’-C5′) angles of maltotriose in our structure are φ = 107.5°, Ψ = −144.3° and φ = 90.8°, Ψ = −153.7°. The first φ/Ψ angles that is near the end of the chain is more obtuse, whereas the φ/Ψ angles cloistered within the binding cleft are similar to those found in double-helical amylose (45). In contrast to Sas20d1, the architecture of the Sas20d2-binding site suggests to us a preference for helical regions within α-glucan.

### Sas20d1 binds to extended α-glucan structures

We used ITC to quantify the affinity of maltotriose, maltoheptaose, and solubilized corn and potato amylopectin binding to the domains of Sas20 and the Sca5X25-2 (Table 3 and Figs. S4–S8). Sas20d1 binds to maltoheptaose (*K*_*d*_ = 1.5 ± 0.3 μM) with a *K*_*d*_ nearly two orders of magnitude stronger than maltotriose (*K*_*d*_ = 187.9 ± 58.1 μM). While the crystal structure revealed a short binding platform for three glucose residues, the enhanced affinity of maltoheptaose suggests that our crystal structure does not capture all possible interactions between the protein and ligand (40). As mentioned earlier, we determined a crystal structure of Sas20d1 with maltoheptaose but did not observe additional density at the nonreducing end beyond that of the maltotriose structure. We did note some fading density toward the reducing end that is directed outside the binding cleft, supporting a lack of specific interaction with the protein at this end. Manual inspection and modeling of an additional glucose at the nonreducing end that is tucked within the binding cleft revealed that Sas20d1 can accommodate a longer ligand here, though there is somewhat more space if modeled in the native structure (Fig. S9, *A*–*C*). We did not observe an additional aromatic residue within this cleft, however, that might provide a platform for an additional glucose. An intermediate conformation of the helices between the maltotriose-bound and native Sas20d1 structures may lead to additional protein–ligand interactions that support maltoheptaose binding, although we could not capture this binding *in* *crystallo*. Regardless, the structure with maltotriose suggested that this domain has some specific preference for binding at the nonreducing ends of starch and maltooligosaccharides. This may in part account for the apparent lack of binding in affinity PAGE with amylopectin, as there is a very low concentration of polymer ends in a high–molecular weight polysaccharide (molecular weight [MW] = ∼10^8^ Da) (48). However, we found that Sas20d1 binds to both corn (*K*_*d*_ = 10.0 ± 1.7 μM) and potato amylopectin (*K*_*d*_ = 17.6 ± 7.2 μM), demonstrating a slight preference for corn amylopectin (Table 3). Therefore, it is likely that some aspect of the affinity PAGE assay was incompatible with Sas20d1 starch binding.

**Table 3:** Affinity of Sas20 and Sca5 constructs for starch substrates determined by ITC

Protein	Ligand	N (binding sites)	*K*_*d*_ (μM)
Sas20d1	Maltotriose	1.14 ± 0.28	187.9 ± 58.1
	Maltoheptaose	0.89 ± 0.38	1.53 ± 0.34
	β-Cyclodextrin	NB	NB
	α-Cyclodextrin	NB	NB
	PNP-M6	1.15 ± 0.07	0.87 ± 0.48
	B-PNP-M7	1.28 ± 0.29	7.12 ± 1.53
	Corn amylopectin	1∗	10.0 ± 1.74
	Potato amylopectin	1∗	17.6 ± 7.18
Sas20d1 Y60A	Maltotriose	NB	NB
	Maltoheptaose	1.55 ± 0.18	8.29 ± 0.51
Sas20d1 W72A	Maltotriose	NB	NB
	Maltoheptaose	NB	NB
Sas20d1tr	Maltotriose	1∗	>1000∗
	Maltoheptaose	1.45 ± 0.27	154.9 ± 63.0
	β-Cyclodextrin	1∗	1050 ± 168
	α-Cyclodextrin	NB	NB
Sas20d2	Maltotriose	1.18 ± 0.05	912.4 ± 110
	Maltoheptaose	1.15 ± 0.15	0.61 ± 0.03
	Corn amylopectin	1∗	7.86 ± 1.4
	Potato amylopectin	1∗	5.68 ± 1.5
	β-Cyclodextrin	0.98 ± 0.09	532.7 ± 16.27
	α-Cyclodextrin	NB	NB
Sas20d2 W329A	Maltotriose	NB	NB
	Maltoheptaose	1.33 ± 0.13	90.84 ± 25.7
Sas20d2 W375A	Maltotriose	NB	NB
	Maltoheptaose	1.12 ± 0.41	88.07 ± 36.0
Sas20d2 W440A	Maltotriose	NB	NB
	Maltoheptaose	1.39 ± 0.37	89.99 ± 7.72
Sas20d2 W481A	Maltotriose	NB	NB
	Maltoheptaose	NB	NB
Sca5X25-2	Maltotriose	1.02 ± 0.62	595.8 ± 51.4
	Maltoheptaose	0.81 ± 0.09	0.21 ± 0.029
	β-Cyclodextrin	0.958 ± 0.01	346.4 ± 78.8
	α-Cyclodextrin	NB	NB
Sca5X25-2a	Maltotriose	NB	NB
	Maltoheptaose	NB	NB
Sca5X25-2b	Maltotriose	NB	NB
	Maltoheptaose	NB	NB

Abbreviations: B-PNP-M7, PNP-α-maltoheptaose with a 4,6-linked-O-benzylidine group at the nonreducing end; NB, no binding detected; PNP-M6, PNP-α-maltohexaose.

Asterisk denotes fixed N or *K*_*d*_. Each N and *K*_d_ are the average of three replicates. Data were fit to a one-site binding model. For polysaccharide titrations, binding is based on the concentration of binding sites.

Sas20d1 failed to bind α-cyclodextrin or β-cyclodextrin supporting our observation that binding is restricted to chain ends. Indeed, when we attempted to model α-cyclodextrin on top of the maltotriose in our structure, there was steric clashing with W205 from the helical bundle (Fig. S9*D*). To test whether the nonreducing ends of maltooligosaccharides are required for binding, we tested binding to benzylidene-blocked para-nitrophenyl maltoheptaoside (B-PNP-maltoheptaose), which has a para-nitrophenyl (PNP) group at the reducing end and 4,6-linked-O-benzylidine at the nonreducing end. We also tested a PNP-α-maltohexaose, which has an exposed O4 at the nonreducing end. Surprisingly, Sas20d1 bound both ligands with a similar *K*_*d*_ as maltoheptaose, though B-PNP-maltoheptaose bound with slightly less affinity (Table 3). Therefore, while our structural and biochemical data support that binding by Sas20d1 is likely limited to chain ends, there is indeed some flexibility within the binding cleft to accommodate a blocked nonreducing end. Specific recognition of the nonreducing end O4 by Sas20d1 is not required for binding.

To further examine the nature of Sas20d1 binding, we created single mutants Y60A and W72A. The Y60A Sas20d1 mutant binds to maltoheptaose but not maltotriose, whereas the W72A mutant did not bind either ligand. This suggests that W72, which is positioned at the reducing end of the binding platform, is required to anchor maltooligosaccharides and perhaps aids in guiding the nonreducing end of the ligand into place. Y60 creates a platform for binding the aglycone face of the nonreducing end glucose and is clearly essential for shorter oligosaccharides, perhaps because these are wedged further within the binding cleft and therefore are not stabilized by interaction with W72. Y60 is not required for maltoheptaose binding which further suggests that there may be additional interactions between ligand and protein that extend beyond the nonreducing end of maltotriose in our structure, but they are difficult to predict from the current models (Fig. S9).

### C-terminal helices are important for substrate binding in Sas20d1

Although the helical bundle at the C terminus of Sas20d1 does not directly interact with maltooligosaccharide, we hypothesized that its presence is an important feature that either lends structural stability to the binding pocket or restricts the binding of cyclodextrins. A truncated version of Sas20d1 lacking these helices (Sas20d1tr, Fig. 1*A*) displayed dramatically reduced binding for maltotriose that could not be quantified *via* ITC, while binding for maltoheptaose decreased by ∼100-fold (Table 3). This truncation did not facilitate binding of α-cyclodextrin or β-cyclodextrin at relevant biological levels (*K*_*d*_ >1 mM). We therefore speculate that these helices support competent binding by providing stability to loops A and B (Fig. 2*D*).

To test if the helices have more order in solution when Sas20d1 is bound to substrate, CD was performed on Sas20d1 alone or with maltotriose or maltoheptaose (Table S2 and Fig. S10*A*). However, there was no significant shift in secondary structure in the presence or the absence of substrate. We then tested if WT Sas20d1 could resist thermal unfolding compared with the Sas20d1tr construct (Table S3, Fig. S10, *B* and *C*). As expected, we observed a marked decrease in α-helical quality in Sas20d1tr compared with the full-length domain. However, the percentage of unordered region remained the same across both Sas20d1 and Sas20d1tr at all temperatures suggesting that the C-terminal helices in Sas20d1 contribute marginally to the stability of this domain.

### Sas20d2 binds to starch

Like Sas20d1, Sas20d2 binds to maltoheptaose (*K*_*d*_ = 0.61 ± 0.03 μM) with greatly enhanced affinity over maltotriose (*K*_*d*_ = 912.4 ± 110 μM), suggesting that the domain utilizes the extensive binding platform between both X25 lobes. Sca5X25-2 shows a nearly identical trend, although the binding for each ligand is modestly better compared with Sas20d2. The number of binding sites (N) for these interactions is ∼1 suggesting that there is only one extended ligand-binding site as observed in the Sca5X25-2 crystal structure. Although each module of Sca5X25-2 resembles a fully competent starch-binding site akin to those found within SusF (Fig. 3), individual constructs of Sca5X25-2a and Sca5X25-2b (Fig. 1*B*) failed to bind either maltotriose or maltoheptaose underscoring the need for the extended platform comprised of four tryptophan residues between both X25s for the high-affinity binding as observed with maltoheptaose.

Neither Sas20d2 nor Sca5X25-2 bound to α-cyclodextrin, but they did bind β-cyclodextrin, albeit with low affinity (∼100-fold higher *K*_*d*_ compared with maltoheptaose), likely because of the increased ability of β-cyclodextrin to contort to a favorable binding geometry (Table 3). Cyclodextrins are often used as a proxy for the recognition of internal regions of a starch polymer, and many starch-binding CBMs recognize cyclodextrins and starch *via* a shallow cleft comprised of two aromatic residues that mimic the curvature of the α1,4-glucan bond (49, 50). While the volume of the Sas20d2-binding site is large enough to accommodate α-cyclodextrin, the helical arrangement of the aromatic platform likely prevents productive binding of the ligand. We quantified our affinity PAGE results (Figs. 1 and S1) by ITC (Table 3) and determined that Sas20d2 binds to both corn (*K*_*d*_ = 7.9 ± 1.4 μM) and potato amylopectin (*K*_*d*_ = 5.7 ± 1.5 μM) with similar affinity. Sas20d2 binds only modestly better to these polysaccharides compared with Sas20d1.

As with Sas20d1, we mutated the four Trp residues (W329A, W375A, W440A, and W481A) in Sas20d2 that corresponded to the aromatic platform observed within the Sca5X25-2 structure (Figs. 3*E* and S3). A consistent trend for each mutation was the loss of binding for maltotriose. This was true for both W440A and W375A, equivalent to W620 and W555 of Sca5X25-2, positioned at the edges of the binding pocket, which we thought might be unnecessary for the smaller ligand. In fact, W555 of Sca5X25-2 (W375 of Sas20d2) did not participate in binding in our crystal structure. W481 of Sas20d2 (W661 of Sca5X25-2) is positioned toward the interior of the binding cavity, and mutation eliminated binding to both maltotriose and maltoheptaose, whereas the W329A, W375A, and W440A mutants retained binding to maltoheptaose but displayed ∼100-fold increase in the *K*_*d*_ compared with WT Sas20d2. Notably, despite the symmetry within the binding pocket, mutations within each lobe had unique phenotypes. Particularly, W481 of the second X25 module seems to be most essential for anchoring maltooligosaccharides. Together, these data underscore that this domain is tuned to recognize longer helical regions of α-glucan including those within the crystalline regions of starch granules.

### Sas20 domains bind to insoluble corn starch

**Figure 4:**
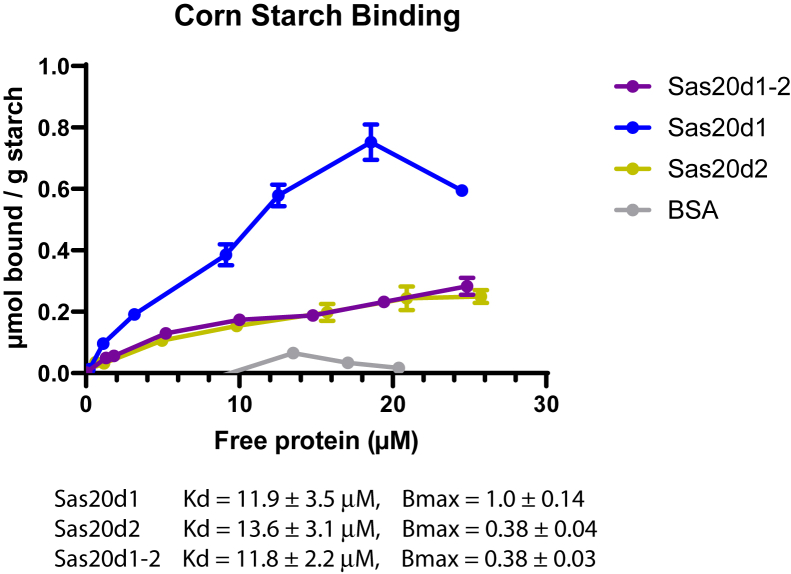
**Isothermal depletion for corn starch.** Affinity by indicated protein constructs on insoluble corn starch. All data fit to a one-site specific binding isotherm model; the *R*^2^ for these curves for Sas20d1, Sas20d2, and Sas20d1-2 is 94.0%, 96.1%, and 96.5%, respectively. Sas20d1, domain 1 of Sas20; Sas20d2, domain 2 of Sas20.

The ITC results allowed us to make conclusions on the binding profile of soluble substrates, but since *R. bromii* degrades RS, we investigated insoluble starch binding of Sas20 to corn starch. Sas20d1, Sas20d2, and Sas20d1-2 had similar *K*_*d*_ values ranging from 10 to 15 μM (Fig. 4). However, Sas20d1 had a *B*_max_ that is nearly triple that of Sas20d2 or Sas20d1-2. This suggests that Sas20d1 can access more binding sites on the corn starch granule. Interestingly, we did not observe synergy or enhanced binding of the protein when both domains were present. This could be because the Sas20d1-2 construct is bulkier, and since each binding site is tuned to recognize different aspects of the polysaccharide, the larger protein makes fewer productive interactions with the granule. Therefore, the sequential position of both domains appears to not display avidity with respect to binding to ligand.

### Sas20 domains are flexible and extended in solution

To better connect how our crystal structures correlate to the substrate preferences we observe in solution, we used size-exclusion chromatography (SEC) coupled with SAXS on Sas20d1, Sas20d2, and Sas20d1-2 with and without 5 mM maltoheptaose (Table S4). Since Sas20d2 could not be crystallized, we used Phyre2 to generate a Sas20d2 model (100% confidence) using the Sca5X25-2 crystal structure for fitting the solution data (51).

**Figure 5:**
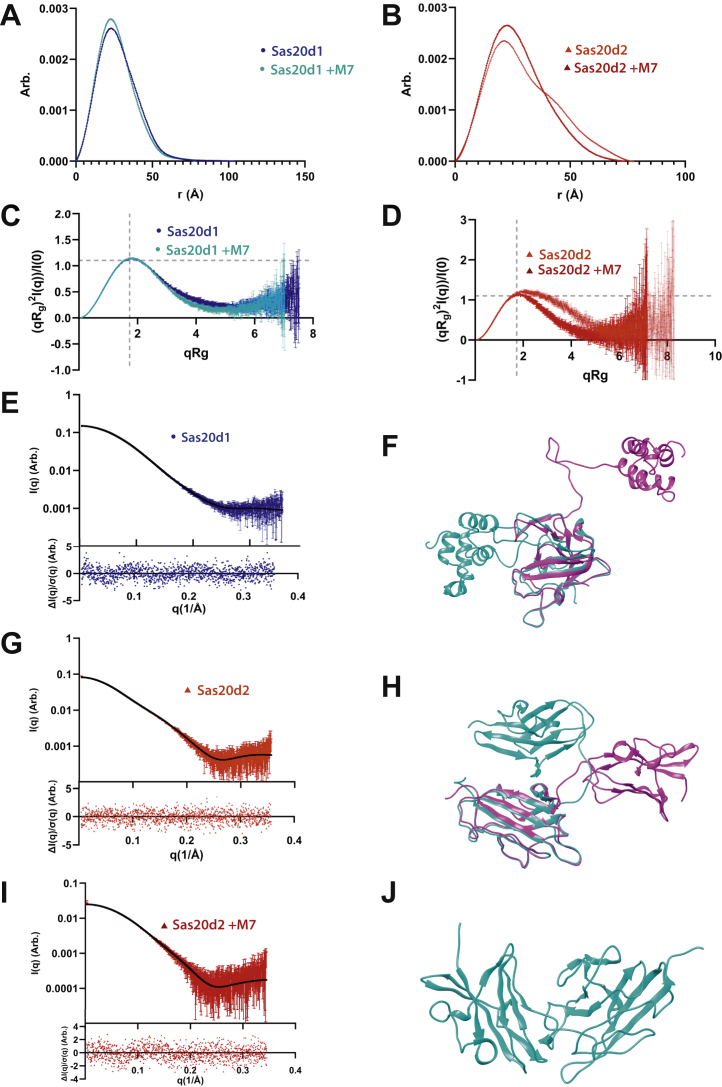
**Experimental SAXS and MultiFoXS results for Sas20d1 and Sas20d2.** Sas20d1 is in *blue circles*, and Sas20d2 is in *red triangles*. P(*r*) *versus r* for (*A*) Sas20d1 and (*B*) Sas20d2 with and without maltoheptaose normalized by *I*(0). Dimensionless Kratky plot for (*C*) Sas20d1 and (*D*) Sas20d2 with and without maltoheptaose; *y* = 3/*e* and $x=\sqrt{3}$ as *dashed gray lines* to indicate where a globular protein would peak. *E*, SAXS scattering profile (*points*) and MultiFoXS fit (*black line*) for Sas20d1 (χ^2^ = 1.19). The *bottom panel* shows the normalized fit residual. *F*, MultiFoXS two-state model results for Sas20d1 with compact (*cyan*, *R*_g_ = 19 Å, weight = 86%) and extended (*magenta*, *R*_g_ = 25 Å, weight = 14%) conformations. Models aligned to residues 32–163 and were slightly offset for clarity. SAXS scattering profile (*points*) and MultiFoXS fit (*black line*) for (*G*) Sas20d2 (χ^2^ = 0.97) and (*I*) Sas20d2 with 5 mM maltoheptaose (χ^2^ = 1.01). The *bottom panel* shows the normalized fit residual. *H*, MultiFoXS two-state model results for Sas20d2 with compact (*cyan*, *R*_g_ = 20 Å, weight = 36%) and extended (*magenta*, *R*_g_ = 24 Å, weight = 64%) conformation. *J*, MultiFoXS one-state model for Sas20d2 with maltoheptaose (*R*_g_ = 19.5 Å). Sas20d1, domain 1 of Sas20; Sas20d2, domain 2 of Sas20; SAXS, small-angle X-ray scattering.

The SEC–SAXS experiments for Sas20d1 and Sas20d2 with and without maltoheptaose were monodisperse, and the radius of gyration (*R*_g_) across the eluted peak was relatively constant (Table 4 and Fig. S11, *A*–*D*). The Guinier fit for the *R*_g_ and *I*(0) values confirmed that these samples were monodisperse (Fig. S12, *A*–*D*). The MWs of Sas20d1 and Sas20d2 with and without maltoheptaose were calculated to be ∼26 kDa, which corroborates the predicted monomeric MW based on their sequences (Table 4). The *D*_max_ values from the P(*r*) function for Sas20d1 without and with maltoheptaose are 103 and 78 Å, respectively, and for Sas20d2 without and with maltoheptaose are 78 and 74 Å, respectively, while the maximum dimension in the crystal structure or model for both proteins are approximately 66 Å (Table 4, Figs. 5, *A* and *B*, S13, *A*–*D*). Together, this suggests that Sas20d1 undergoes a contraction upon the addition of ligand, whereas only a marginal contraction occurs with Sas20d2. In addition, the calculated *D*_max_ indicates that Sas20d1 and Sca5X25-2 were crystallized in a relatively compact conformation in contrast to their average conformation in solution.

**Table 4:** Small-angle X-ray data

Protein	*I*(0)	*R*_g_ (Å) SAXS	*D*_max_ (Å) crystal	*D*_max_ (Å) solution	Sequence MW (kDa)	SAXS MW (kDa)
Sas20d1	1.5 × 10^−6^ ± 6.0 × 10^−10^	21.1 ± 0.02	64.3	103	25.9	25.6
Sas20d1 + maltoheptaose	0.05 ± 2.3 × 10^−5^	20.4 ± 0.03	60.6	78		24.3
Sas20d2	8.3 × 10^−7^ ± 5.3 × 10^−10^	23.1 ± 0.04		78	26.5	25.6
Sas20d2 + maltoheptaose	0.03 ± 2.6 × 10^−5^	20.8 ± 0.04	67.5	74		25.9
Sas20d1-2	0.04 ± 7.9 × 10^−5^	53.9 ± 0.26		203	57.2	46.6
Sas20d1-2 + maltoheptaose	0.04 ± 6.4 × 10^−5^	51.8 ± 0.17		190		53.1

*I*(0) and *R*_g_ were determined from Guinier analysis. *D*_max_ in solution was determined by indirect Fourier transform using GNOM. To calculate *D*_max_*incrystallo*, we calculated the farthest distance between two amino acids in one peptide in the crystal structures for native Sas20d1, maltotriose-bound Sas20d1, and Phyre 2.0-generated model for Sas20d2. The Bayes method of molecular weight calculation from SAXS data is presented here.

The overall shape of the P(*r*) function for Sas20d1 and Sas20d2, calculated by indirect Fourier transform using GNOM (52), has a relatively Gaussian shape that is characteristic of a globular compact particle (Fig. 5, *A* and *B*). Upon the addition of ligand, the P(*r*) function demonstrates that Sas20d1 undergoes a contraction in solution, but the overall shape of the P(*r*) function, and thus the protein itself, remains relatively constant. There is a truncation in the tail of the function, which can be interpreted as a decrease in flexibility upon binding to ligand. However, the P(*r*) function for Sas20d2 without ligand shows a clear shoulder near *r* = 40 Å, which is characteristic of a protein with two structural motifs. This right shoulder is not found in the presence of ligand, which suggests that the two lobes seen in Sas20d2 associate more tightly upon binding to ligand while retaining the overall size of the protein.

The dimensionless Kratky plot maxima for Sas20d1 and Sas20d2 are where typical rigid globular proteins would peak (Fig. 5, *C* and *D*). Upon addition of maltoheptaose, Sas20d1 shows a small but significant decrease in the mid-to-high q region, around q*R*_g_ = 4, which indicates the ligand made this protein more compact and globular in solution. In the Sas20d2 analysis, the small plateau in the mid-to-high q region, around q*R*_g_ = 4 in the dimensionless Kratky plot, indicates some extension or flexibility in the system, likely associated with the two structural motifs visible *via* the P(*r*) plot. This plateau vanishes in the presence of maltoheptaose, and the resulting dimensionless Kratky plot shows that the protein with ligand is a more compact globular shape. Thus, the SAXS shows that ligand binding results in a more compact, globular shape of Sas20d2.

To fit our high-resolution structures to the SAXS data, we used MultiFoXS (multistate modeling with SAXS profiles) to generate a set of possible conformations in solution and selected the ensemble with the best fit (53). For Sas20d1, we assigned the linker between the CBM26-like structure and bundle of helices (residues 164–191) as flexible. Since the differences in the basic SAXS analysis were subtle, MultiFoXS modeling was only done for Sas20d1 without ligand. MultiFoXS found that the best-fit solution was with two states, one compact and one extended with a χ^2^ = 1.19 (Fig. 5, *E* and *F*). Sas20d1 only exists in the extended conformation ∼14% of the time in solution, which agrees with the compactness and minimal flexibility indicated by the P(*r*) distribution and dimensionless Kratky plot.

Since the differences in the basic SAXS analysis indicated that there was a significant change in shape upon addition of ligand to Sas20d2, MultiFoXS modeling was done for both Sas20d2 with and without ligand. We assigned the linker between the two X25-like lobes (residues 415–423) as flexible. For Sas20 without ligand, MultiFoXS found that the best-fit solution was also with two states, one compact and one extended with a χ^2^ = 1.01 (Fig. 5*G*). In contrast to Sas20d1, Sas20d2 without ligand exists in the extended state ∼64% of the time in solution (Fig. 5*H*). When ligand is present, MultiFoXS found the best-fit solution was a one-state model that resembles the compact conformation (Fig. 5, *I* and *J*). Both ensembles corroborate the shapes indicated by the P(*r*) function and Kratky plots. However, because there is flexibility in the system, the displayed states in Figure 5, *F*, *H*, and *J* are representative of these extended and compact conformations but should not be taken as prescriptive; that is, there are likely many similar states with the same overall size and extension but slightly different relative positions of the two folded motifs.

**Figure 6:**
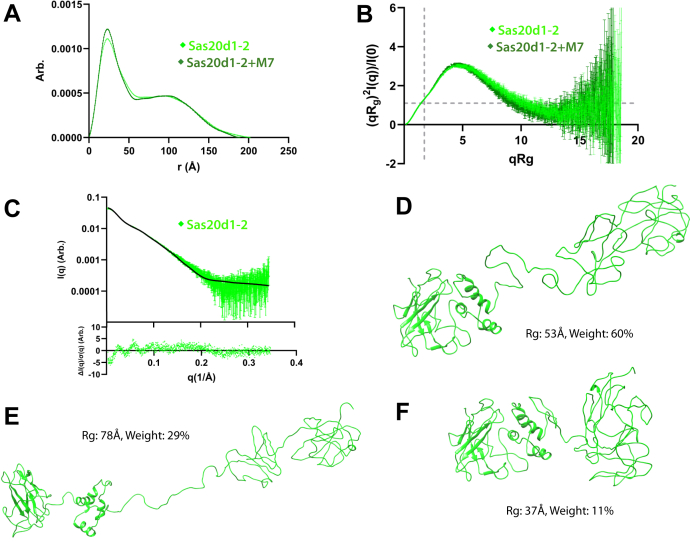
**Experimental SAXS and MultiFoXS results for Sas20d1-2**. Sas20d1–2 in *green diamonds*. *A*, P(*r*) *versus r* for Sas20d1-2 and Sas20d1-2 with maltoheptaose normalized by *I*(0). *B*, dimensionless Kratky plot with *y* = 3/*e* and $x=\sqrt{3}$ as *dashed gray lines* to indicate where a globular protein would peak. *C*, the SAXS scattering profile (*green points*) and MultiFoXS fit (*black line*) for Sas20d1-2 (χ^2^ = 2.65). The *bottom panel* shows the normalized fit residual. *F*–*H*, MultiFoXS three-state results for Sas20d1-2 with their associated *R*_g_ and weight. SAXS, small-angle X-ray scattering.

We then performed SEC–SAXS on Sas20d1-2 with and without 5 mM maltoheptaose to discern how the two domains are oriented in solution and if this protein possesses notable flexibility. The elution profiles revealed that the SEC column separated a minor contaminant (peak 1520 s) in the Sas20d1-2 run and two minor contaminants (peaks 1650 and 2050 s) from the Sas20d1-2 with maltoheptaose run from our protein of interest (peak, 1370 s) (Fig. S11, *E* and *F*). The *R*_g_ across the eluted peaks was relatively constant. The Guinier fit for the *R*_g_ and *I*(0) values confirmed that Sas20d1-2 with and without maltoheptaose were monodisperse (Fig. S12, *E* and *F*). The calculated MW from the scattering profile, 53.7 kDa, agreed with the predicted monomeric MW by sequence (Table 4). The right shoulder in the P(*r*) plot is characteristic of a second domain with significant (∼100 Å) separation from the first and is consistent with some flexibility given the long tail down to the maximum dimension of ∼200 Å (Figs. 6*A*, S13, *E* and *F*). The shape of the dimensionless Kratky plot for Sas20d1-2 shows significant deviation from where we expect globular proteins to peak (Fig. 6*B*). In particular, the peak near q*R*_g_ of 5 is above 2, which indicates a highly extended molecule, and the plateau at higher q*R*_g_ also indicates some flexibility in the system. As with Sas20d1, addition of maltoheptaose to Sas20d1-2 had a subtle effect on the overall shape of the protein but induced a more globular shape and decrease in flexibility.

We then used MultiFoXS with our high-resolution structure of the Sas20d1 domain and model of Sas20d2 in isolation to investigate how the domains are positioned relative to each other. The best model fit was a three-state ensemble with an acceptable χ^2^ = 2.65, but the residual from this fit to the SAXS scattering profile is not randomly distributed, particularly in the low q range (Fig. 6*C*). Here, we see that Sas20d1-2 shows a range of conformations from very compact to very extended, where this protein exists in the most compact state only ∼11% of the time (Fig. 6, *D*–*F*). This agrees with the observations from the P(*r*) function and dimensionless Kratky plot, which showed highly extended flexible systems with well-separated domains. Also, no single solution, compact or extended, fits the data well, as the best single model fit has a χ^2^ = 8.2, further indicating a flexible system that exists in a continuum of states in solution. In conclusion, while the precise number and extent of conformations adopted by Sas20d1-2 in solution is unclear, both the MultiFoXS and basic SAXS analysis indicate that Sas20d1-2 is highly flexible and extended in solution.

### Sas20 domain homology

Sas20 has two distinct domains that recognize different aspects of the starch substructure. To determine if the Sas20 domains occur in other bacteria, we performed a BLAST analysis of each Sas20 domain (54). Using an *E* value <0.01, we found 101 sequences for the first domain, and the vast majority of these are found within *Ruminococcus* species, suggesting an extremely narrow phylogenetic distribution (Fig. S14). Among these sequences, many possess homology to domain 1 and Sas20d2. Interestingly, we discovered that *R. bromii* has a second Sas20d1-like protein. The protein encoded within locus tag RBR_02940 (L2-63_00923) of *R. bromii* L2-63 is a predicted cell wall–anchored protein and shares 31% sequence identity with Sas20d1 along the length of the β-sandwich and including part of the α-helical bundle. Using JPred4 for secondary structure prediction, RBR_09240 is expected to possess four helices that are C terminal to the β-sandwich and followed by a Gly-Ser-Asn–rich linker and sortase motif (Fig. S15) (55). Most of the maltotriose-binding platform observed in the Sas20d1 structure is conserved in RBR_09240, except for Y60 (substituted conservatively as tryptophan) and T152 (substituted for proline). Therefore, we predict that RBR_09240 is a starch-binding cell surface–anchored protein but is unlikely to be incorporated into an amylosome complex because of its apparent lack of a dockerin or cohesin module. Interestingly, the genomic context for this protein does not further imply function, as the gene is sandwiched between a predicted alanine-tRNA ligase and probable endonuclease.

Like Sas20d1, Sas20d2 is fairly restricted in its phylogenetic distribution. We found 328 sequences with homology to Sas20d2 *via* BLAST (E value <0.0001), of which 206 were from *Ruminococcus*, 24 from the CFB bacteria (Cytophaga–Fusobacterium–Bacteroidetes), and the remainder within the Firmicutes, many in the Oscillspiracaea, which includes *Ruminococcus*. Of the 328 sequences, only 19 were identified by the DBCan server as sharing homology with a known CBM or glycoside hydrolase family; 12 of these proteins appear to possess multiple starch-targeting CBMs and/or a GH13 in addition to a domain with homology to Sas20d2 (Fig. S16) (56). Most of these sequences retain the residues found in Sca5X25-2 that are involved in capturing maltooligosaccharide (Fig. S17). Beyond Sca5 and Sas20, the scaffoldin protein Sca3 of *R. bromii* L2-63 is predicted to consist of four X25-like modules (13). However, a sequence alignment of the Sca3 domains with the X25s within Sca5 and Sas20 suggests that only one tryptophan is conserved (Fig. S18). Sca3 may bind starch, but the sequence diverges from what is seen in Sca5 and Sas20.

## Discussion

We harnessed a diverse array of biophysical and biochemical techniques to perform a structure–function characterization of Sas20, a multidomain starch-binding amylosome protein in *R. bromii*. Our data revealed that one of these domains, Sas20d1, seems to have a binding preference for the nonreducing ends of starch chains. In plants, starch granules are synthesized as a series of concentric layers of amorphous and semicrystalline regions of amylose and amylopectin, from the reducing to the nonreducing end. The reducing ends of the α-glucan chains in amylopectin are less accessible as they are involved in the α1,6 glycosidic linkage that creates the branch points in amylopectin, whereas the nonreducing ends are much more abundant within these layers (57). Because of the way starch is synthesized in plants, nonreducing ends may be more enriched toward the surface of the granules, and Sas20d1 may aid in anchoring *R. bromii* to the starch granule surface (57, 58, 59). The Sas20d1 with maltotriose crystal structure showed a closing in of the bundle of two loops and α-helices over the ligand (Fig. 2, *D* and *F*), representative of the more compact states of Sas20d1, compared with the more extended states observed *via* SAXS (Fig. 5). It is possible that the apparent ability of the Sas20d1 site to open facilitates the capture of the ends of the α-glucan chains within starch granules. The geometry of this binding site, based upon the orientation of maltotriose in the crystal structure, seems to not only target the nonreducing end of the α-glucan but favors a somewhat less helical α1,4-linked chain as might be more thermodynamically feasible at the chain end. Despite our belief that the data largely support the model that binding is favored at the nonreducing end of the α-glucan chain, we cannot completely exclude that Sasd1 may also recognize interior regions of the polysaccharide, perhaps *via* one of its more extended conformations.

In contrast to Sas20d1, Sas20d2 has an elongated binding platform created by two X25 modules in tandem, which create a clamshell-type structure that can recognize the helical turn of the α1,4 glycosidic bond. This binding site features four tryptophan residues, which is more extensive than the typical dual aromatic amino acid motif found in most structurally characterized starch-binding CBMs (19). While the individual X25 modules of proteins, such as SusE and SusF, which have two and three X25s, respectively, bind maltooligosaccharides, our constructs of the individual X25 modules from Sas20d2 failed to demonstrate maltooligosaccharide binding (47). Sca5X25-2 and Sas20d2 demonstrate a ∼1500× lower *K*_*d*_ for maltoheptaose over maltotriose, a modest preference for the longer sugar, similar to what we observed with Sas20d1 binding for these same substrates. For Sas20d2, the participation of both X25 modules in binding may be required to close the protein around the helical ligand, as suggested by the SAXS analysis of the domain with and without ligand. Sas20d2 failed to bind α-cyclodextrin and demonstrated weak binding for β-cyclodextrin, which supports that the specific helical geometry of starch is indeed recognized, likely imposed by arrangement of the elongated binding platform.

In our isothermal depletion experiments, all constructs had similar affinities to starch granules, underscoring that both domains, despite the differences in their architectures, contribute to starch binding. We were somewhat surprised that Sas20d1-2 had a lower *B*_max_ than Sas20d1 on insoluble corn starch, as we speculated that additional binding modules may allow the protein to find more binding sites on the granule. It seems that instead the larger two-domain construct binds to fewer places on the granule, perhaps because the two domains recognize different structural motifs and/or the larger protein is more sterically restricted from adopting a range of binding orientations with the granule. Sas20, as part of cell-surface amylosomes, may provide the flexible recognition of different aspects of the starch structure that are revealed during RS degradation. The ability to recognize different parts of starch may be important for efficient RS degradation and may be one reason why there are several genes encoding putative starch-binding/dockerin-containing proteins in the *R. bromii* genome (14).

The SAXS data revealed that both Sas20 domains are flexible and less compact in solution compared with the crystal structure and homology model. However, contraction was observed in all samples in solution upon binding to ligand, especially Sas20d2. Because each individual domain displays a significant amount of flexibility, it is difficult to determine how the linker contributes to this in the full-length construct, though, presumably this linker adds to the potential range of conformations of the protein in solution which may enhance the ability of the protein to find starch motifs. Linkers between cellulose-active domains in the cellulosome have significant impacts on the higher-order structure of these complexes. Modifications and characteristics like heavy glycosylation, increased concentration of glycines, or negative charged amino acids, and even short disulfide-bridged loops may contribute to the extension of these complexes (60, 61, 62, 63). The linker between Sas20d1 and Sas20d2 is threonine rich and may be a target of *O*-glycosylation; however, there are no data about protein glycosylation in *R. bromii* to date. Since our recombinant protein work was expressed in *Escherichia coli* which lacks the machinery required for *O*-glycosylation of proteins, it is still unclear if this linker is indeed glycosylated and how that modification affects the extension of Sas20.

**Figure 7:**
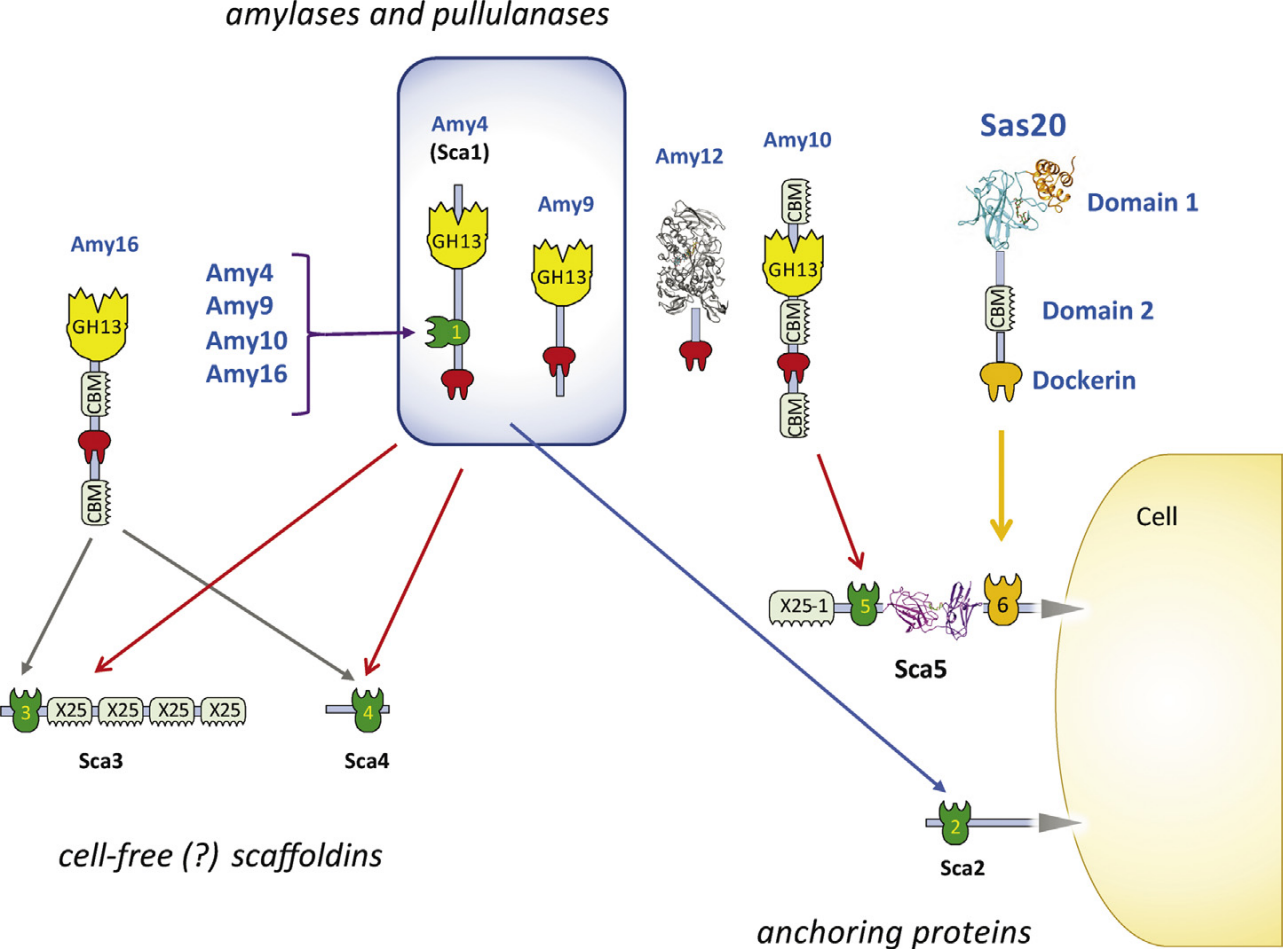
**Updated model for cell-bound and cell-free amylosome complexes in *Ruminococcus bromii* L2-63.** We have added our newly found cohesin–dockerin interaction between the Sas20 dockerin and second cohesin of Sca5 to the most recent model of the amylosome system in *R. bromii*, adapted from the study by Mukhopadhya *et al.* (14). The crystal structures solved of amylosome protein domains in Sca5, Sas20, and Amy12 (PDB: 7LSA) are shown (64). PDB, Protein Data Bank; Sas20, starch adherence system protein 20.

With our data on Sas20, we present an updated model of the known cohesin–dockerin interactions that make the amylosome system (Fig. 7) (13, 14). Previous work and our EDTA elution experiment highlight that there are many other dockerin-containing amylosome proteins that are worthy of biochemical and/or structural characterization (Tables 1 and S1) (14, 64). Equally important to the biochemical properties of the starch-active portions of these proteins are their mechanisms of assembly into their respective amylosome complexes. In the cellulosome system, cohesin–dockerin interactions are important in dictating the final architecture of the complex and even ligand preferences therein (29). Each cohesin–dockerin complex differs in their binding interface, and this interface relates to their role in the cellulosome (65). Moderate-affinity cohesin–dockerin interactions can permit the exchange of dockerin-containing enzymes in the cellulosome depending upon the substrates in the environment (66). This allows enzymes with different substrate preferences to be incorporated into the cellulosome when the cell detects a change in the environmental polysaccharide. However, there is little evidence that genes encoding amylosome proteins are differentially regulated by exposure to different monosaccharides or different forms of starch (13, 67). It is possible that at different phases in *R. bromii* growth, there are subtle changes in amylosome protein composition that may affect the types of amylosomes that are assembled. Therefore, further studies on the Sas20 dockerin and its interaction with the second cohesin of Sca5 are important for understanding the full role of Sas20 in *R. bromii*.

## Experimental procedures

### Growth and proteomic analysis of *R. bromii*

Freezer stocks of *R. bromii* L2-63 were inoculated into 2 × 10 ml RUM medium as described (13) supplemented with 1% galactose or autoclaved potato amylopectin in an anaerobic chamber (85% N_2_, 10% H_2_, and 5% CO_2_) and grown until they reached an absorbance of 0.5 at 600 nm (∼48 h). Aliquots totaling 20 ml from each condition were harvested by centrifugation (4500*g* for 5 min). Cells were resuspended in 1 ml of PBS (137 mM NaCl, 2.7 mM KCl, 10 mM Na_2_HPO_4_, and 1.8 mM KH_2_PO_4_ [pH = 7.4]). The cells were again subjected to centrifugation and resuspended in 400 μl of PBS or PBS with 10 mM EDTA and left to incubate at room temperature for 20 min. The cells were centrifuged again, and the supernatant was stored at −80 °C before proteomic analysis.

### Proteomic analysis

*R. bromii* proteomic analysis was performed at the University of Michigan Proteomics Resource Facility. Cysteines were reduced by adding 10 mM DTT and incubating at 45 °C for 30 min. Samples were then cooled to room temperature, and alkylation of cysteines was achieved by incubating with 65 mM 2-chloroacetamide, under darkness, for 30 min at room temperature. An overnight digestion with sequencing grade–modified trypsin (enzyme:substrate ratio of 1:50) was carried out at 37 °C with constant shaking in a ThermoMixer (Eppendorf). Digestion was stopped by acidification, and peptides were desalted using SepPak C18 cartridges using the manufacturer’s protocol (Waters). Samples were completely dried using a Vacufuge (Eppendorf), and resulting peptides were dissolved in an appropriate volume of 0.1% formic acid/2% acetonitrile solution to achieve ∼500 ng peptide/μl. About 2 μl of the peptide solution was resolved on a nanocapillary reverse-phase column (Acclaim PepMap C18, 2 micron, 50 cm; Thermo Fisher Scientific) using a 0.1% formic acid/2% acetonitrile (buffer A) and 0.1% formic acid/95% acetonitrile (buffer B) gradient at 300 nl/min over a period of 90 min (2–25% buffer B in 45 min, 25–40% in 5 min, 40–90% in 5 min followed by holding at 90% buffer B for 5 min and equilibration with buffer A for 30 min). Eluent was directly introduced into an Orbitrap Fusion Tribrid mass spectrometer (Thermo Fisher Scientific) using an EasySpray source. Mass spectrometry 1 (MS1) scans were acquired at 120 K resolution (automatic gain control target = 1 × 10^6^; max injection time = 50 ms). Data-dependent collision-induced dissociation MS/MS spectra were acquired using the Top speed method (3 s) following each MS1 scan (normalized collision energy ∼32%; automatic gain control target = 1 × 10^5^; and maximum injection time = 45 ms). Proteins were identified by searching the data against the *R. bromii* L2-63 protein database with 2111 entries, provided by Dr Paul Sheridan at the Rowett Institute, using Proteome Discoverer (version 2.4; Thermo Fisher Scientific). Search parameters included MS1 mass tolerance of 10 ppm and fragment tolerance of 0.1 Da; two missed cleavages were allowed; carbamidimethylation of cysteine was considered as fixed; oxidation of methionine and deamidation of asparagine and glutamine were considered as potential modifications. False discovery rate was determined using percolator, and proteins/peptides with a false discovery rate of ≤1% were retained for further analysis.

### Cloning, protein expression, and purification

All genes and gene fragments were amplified from *R. bromii* genomic DNA using the Phusion Flash polymerase (Thermo Fisher Scientific) according to the manufacturer’s instructions for ligand-independent cloning with the Expresso T7 Cloning system using the pETite N-His vector kit (Lucigen) according to the manufacturer’s instructions. Primer sequences are listed in Table S5 wherein the N terminus contained a tobacco etch virus protease cleavage site immediately downstream of the complementary 15 bp overlap (encoding the His tag) to create a tobacco etch virus–cleavable His-tagged protein. Site-directed mutagenesis was performed using the Agilent Technologies QuikChange Lightning Site-Directed Mutagenesis Kit according to the manufacturer’s instructions.

For Sas20 dockerin–cohesin interaction studies, the PCR product was digested with KpnI and BamHI restriction enzymes (New England Biolabs, Inc) and inserted into the restricted pET28a, containing *Geobacillus stearothermophilus* xylanase T-6 (39). CBM-Cohs were cloned as described previously (13, 14). All plasmid insert sequences were verified by Sanger sequencing conducted by Eurofins Scientific. Xyn-Sas20 and the CBM-Coh fusion proteins were expressed in *E. coli* BL21 pLysS (DE3) and purified as described by Ben David *et al.* (68). To determine potential Sas20 interactions to *R. bromii* cohesins, the standard affinity-based ELISA procedure of Barak *et al.* (39) was performed.

Expression plasmids were transformed into *E. coli* Rosetta (DE3) pLysS cells, expressed, and purified as previously described (69). Selenomethionine-substituted Sca5X25-2 was produced by first transforming the plasmid into *E. coli* Rosetta (DE3) pLysS and plating onto LB, supplemented with kanamycin (50 μg/ml) and chloramphenicol (20 μg/ml). The bacteria were grown for 16 h at 37 °C, and then colonies were harvested from the plate to inoculate 100 ml of M9 minimal medium supplemented with the same antibiotics. After 16 h of incubation at 37 °C, this starter culture was used to inoculate a 2-l baffled flask containing 1 l of Molecular Dimensions Seleno-Met premade medium, supplemented with 50 ml of the recommended sterile nutrient mix, chloramphenicol, and kanamycin. Cultures were incubated at 37 °C to an absorbance of 0.5 at 600 nm, the temperature was adjusted to 23 °C, and each flask was supplemented with 100 mg each of l-lysine, l-threonine, and l-phenylalanine and 50 mg each of l-leucine, l-isoleucine, l-valine, and l-selenomethionine (70). After 20 min of further incubation, protein expression was induced by the addition of 0.5 mM IPTG, and cultures were allowed to grow for an additional 48 h before harvest by centrifugation. Cells were then lysed by sonication, and the protein purified as previously described *via* nickel affinity chromatography (69).

### Affinity PAGE

Native 10% polyacrylamide gels with and without 0.1% added polysaccharide (glycogen, pullulan, autoclaved potato and corn amylopectin, and dextran) were cast with 0.375 M Tris–HCl (pH 8.8) as described (71). Gels were subjected to 100 V for 4 h and then stained for 2 h with 0.1% Coomassie Brilliant Blue R-250 in 10% acetic acid, 50% methanol, and 40% water before destaining with solution lacking dye overnight with one change of solution.

Binding was considered positive if the migration of the protein in the polysaccharide gel relative to a noninteracting protein (bovine serum albumin) was significantly slower (<0.85 relative mobility) compared with that in the control gel.

### Crystallization and X-ray structure determination

Sas20d1 crystallization experiments were performed using a Crystal Gryphon (Art Robbins) in 96-well trays using a sitting drop format. Diffraction quality crystals of native Sas20d1 were obtained by mixing 35 mg/ml protein 1:1 (v/v) with the crystallization solution containing 0.024 M 1,6-hexanediol; 0.024 M 1-butanol, 0.024 M 1,2-propanediol; 0.024 M 2-propanol; 0.024 M 1,4-butanediol; 0.024 M 1,3-propanediol; 0.1 M imidazole; 0.1 M MES monohydrate (pH = 7.5); 20% PEG 500 monomethyl ether; and 10% PEG 20,000. Native Sas20d1 crystals were plunged directly from the well into liquid nitrogen for X-ray data collection. Sas20d1 (32 mg/ml) plus 10 mM maltotriose was subjected to a series of 24-well hanging-drop sparse matrix screens to identify crystallization conditions. Crystals were obtained *via* hanging-drop vapor diffusion at room temperature against 27% PEG 4000, 0.2 M MgCl_2_, 0.1 M Tris (pH = 7.5). Prior to data collection, crystals were cryoprotected by swiping through a solution of 80% mother liquor supplemented with 20% ethylene glycol and then plunged into liquid nitrogen. Selenomethionine-substituted Sca5X25-2 (40 mg/ml) plus (10 mM) maltotriose was subjected to a series of 96-well hanging-drop sparse matrix screens to identify crystallization conditions. Crystals were obtained *via* hanging-drop vapor diffusion at room temperature against 2 M ammonium sulfate and 0.1 M sodium acetate (pH 4.6). Prior to data collection, crystals were cryoprotected by swiping through a solution of 70% mother liquor supplemented with 30% glycerol and then plunged into liquid nitrogen.

X-ray data from Sas20d1 crystals were collected at the Life Sciences Collaborative Access Team beamline ID-F of the Advanced Photon Source at Argonne National Laboratory, and data from Sca5X25-2 crystals were collected at beamline ID-G from the same source. The Sas20d1 structure was determined *via* sulfur SAD phasing using multiple datasets, processed, and merged within HKL2000 and Scalepack (72), and the maltotriose-bound Sas20d1 structure was phased by molecular replacement with the native Sas20d1 dataset. The Sca5X25-2 with maltotriose structure was phased by selenomethionine substitution. Phasing was performed using AutoSol in Phenix (73). The protein models were finalized *via* alternating cycles of manual model building in Coot and refinement in Phenix.refine and/or Refmac5 from the CCP4 suite (74, 75, 76).

### ITC

ITC measurements were carried out using a TA Instruments Nano ITC. Proteins were dialyzed into 50 mM Hepes (pH = 7.0), and oligosaccharides were prepared using the dialysis buffer. Protein (25–75 μM) was placed in the sample cell, and the reference cell was filled with water. After the temperature was equilibrated to 25 °C, a first injection of 2 μl was performed, followed by 29 subsequent injections of 10 μl of 2 to 10 mM maltotriose, maltoheptaose, or 0.025% autoclaved corn and potato amylopectin. For polysaccharide titrations, the concentration of ligand was adjusted to fit a one-site binding model with n = 1; this sets the concentration of the ligand to the concentration of binding sites for the protein within the polysaccharide, as previously described (77). The solution was stirred at 250 rpm, and the resulting heat of reaction was measured. Data were analyzed using the TA Instruments NanoAnalyze software package fitting to a one-site binding model. Isotherms are displayed in Figs. S4–S8.

### Isothermal depletion assay

Recombinantly expressed protein binding to raw corn starch (National Starch Food Innovation 9735) was determined by adsorption as previously described (47, 77). Raw starch was prepared by washing with sterile PBS three times by resuspension and centrifugation. Aliquots (150 μl) of 10% w/v starch were aliquoted into 0.2 ml tubes, pelleted by centrifugation (2000*g*), and the supernatant fluids were removed leaving 15 mg of raw starch per tube in triplicate for each concentration. Aliquots (150 μl) of protein (0–1.0 mg/ml) in 100 mM NaCl and 20 mM (pH = 7.0) HEPES buffer was added to the starch for a final 10% w/v of starch. Triplicate reactions were agitated by inversion for 1 h at 23 °C and then pelleted (2000*g*), and the protein concentration remaining in the supernatant was measured by Pierce Bicinchoninic Acid assay, using free protein concentrations to create a standard curve for each construct. The results were validated by measuring absorbance at 280 nm on a NanodropC with the theoretical MW and extinction coefficient for each protein. The micromole protein bound was determined by subtracting the bound protein measurement from the free protein value and normalized to the amount of starch as micromole bound per gram of starch. Bovine serum albumin was used as a nonbinding negative control. A one-site specific binding model was used to determine *K*_*d*_ and *B*_max_ in GraphPad Prism (GraphPad Software, Inc).

### CD

Determination of CD spectra for both WT and the truncation mutant was carried out with a J-815 CD spectropolarimeter (Jasco). A protein concentration of 0.1 mg/ml was prepared in 10 mM KH_2_PO_4_ buffer (pH = 7.5). Substrate was added to a concentration of 1 mM and incubated for 24 h with protein before performing CD. A quartz cell with a path length of 0.1 cm was used. Three CD scan replicates per condition were carried out at 25 °C from 190 to 260 nm at a speed of 50 nm/min with a 0.5 nm wavelength pitch. Data files were analyzed with the DICHROWEB online server (http://dichroweb.cryst.bbk.ac.uk/html/process.shtml) using the CDSSTR algorithm with reference set 4, which is optimized for analysis of data recorded in the range of 190 to 240 nm. Mean residue ellipticity was calculated using millidegrees recorded, MW, number of amino acids, and concentration of protein. Temperature interval experiments were performed in triplicate with a protein concentration of 0.1 mg/ml prepared in 10 mM KH_2_PO_4_ buffer (pH = 7.5). CD scans were collected from 190 to 260 nm at a speed of 50 nm/min with a wavelength pitch of 1 nm at temperature intervals of 10 °C between 25 and 95 °C.

### SEC–SAXS experiments

SAXS was performed at BioCAT beamline 18ID at the Advanced Photon Source at Argonne National Labs using in-line SEC–SAXS to separate sample from aggregates and other contaminants. Sample was loaded onto a Superdex 200 Increase 10/300 GL column (Cytiva), which was run at 0.6 ml/min by an AKTA Pure FPLC (GE), and the eluate after it passed through the UV monitor was flown through the SAXS flow cell. The flow cell consists of a 1.0 mm ID quartz capillary with ∼20 μm walls. A coflowing buffer sheath was used to separate sample from the capillary walls, helping prevent radiation damage (78). Scattering intensity was recorded using a Pilatus3 X 1M (Dectris) detector, which was placed 3.6 m from the sample providing a *q* range of 0.005 to 0.35 Å^−1^. Exposures of 0.5 s were acquired every 1 s during elution, and data were reduced using BioXTAS RAW 2.1.0 (79). Buffer blanks were created by averaging regions flanking the elution peak and subtracted from exposures selected from the elution peak to create the *I*(*q*) *versus q* curves used for subsequent analyses. The Bayes method was used to calculate MWs (80). MultiFoXS was used to generate ensembles using the SAXS data and high-resolution crystal structures or models (53).

## Data availability

The MS proteomics data have been deposited to the ProteomeXchange Consortium *via* the PRIDE (81) partner repository with the dataset identifier PXD032013. The X-ray structures and diffraction data reported in this article have been deposited in the PDB under the accession codes 7RPY, 7RFT, and 7RAW. The SAXS data are deposited in the SAXS database under the accession codes SASDMX9, SASDMY9, SASDMZ9, SASDN22, SASDN32, and SASDN42 (82).

## Supporting information

This article contains supporting information (52, 83, 84, 85, 86, 87, 88, 89, 90, 91).

## Conflict of interest

The authors declare that they have no conflicts of interest with the contents of this article.
