# Supplementary material for: Sas20 is a highly flexible starch-binding protein in the Ruminococcus bromii cell-surface amylosome
Source: J Biol Chem. 2022 Apr 1;298(5):101896. doi: 10.1016/j.jbc.2022.101896 (PMC9112005; doi:10.1016/j.jbc.2022.101896)
Supplement: Supplemental Figures S1–S18 [file mmc1.pdf]

A

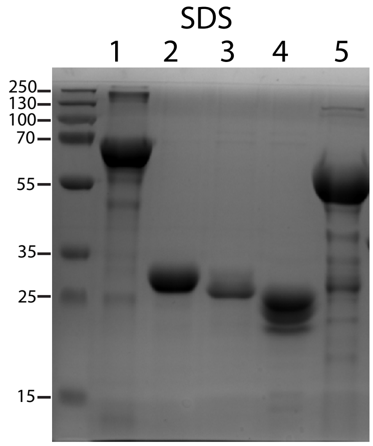

Lanes 1-5 for panels A and B

(1) Bovine serum albumin (BSA)
(2) Sas20d1
(3) Sas20d2
(4) Sca5X25-2
(5) Sas20d1-2

B

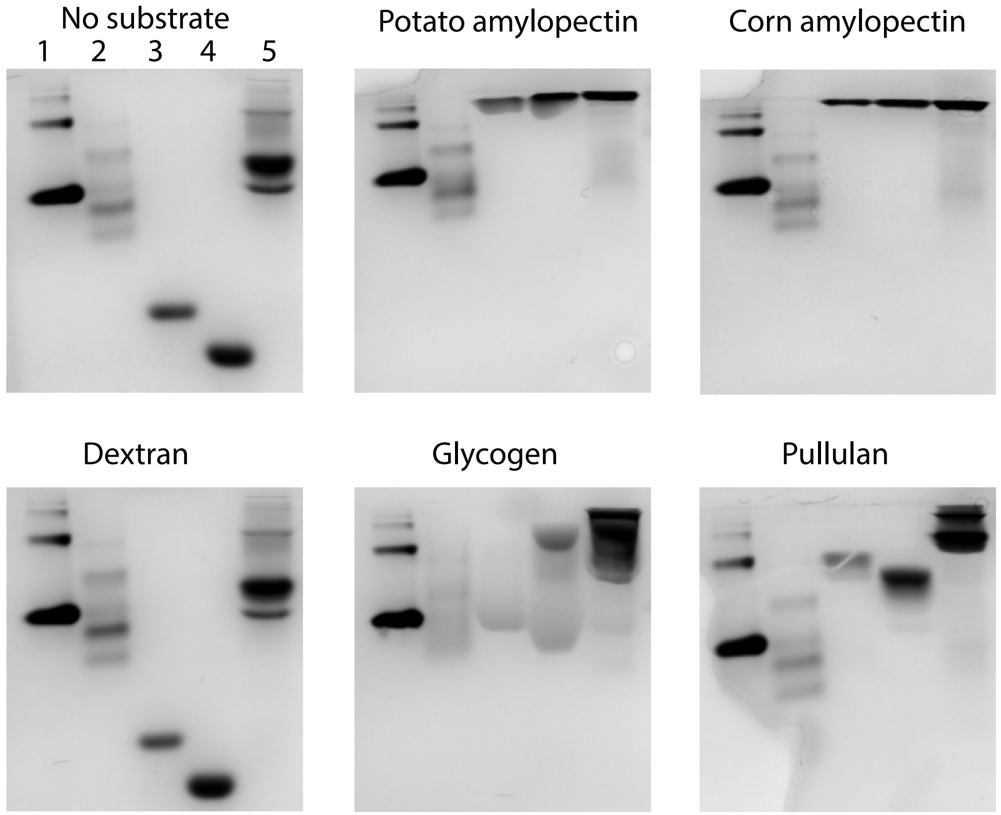

Supplemental Figure 1

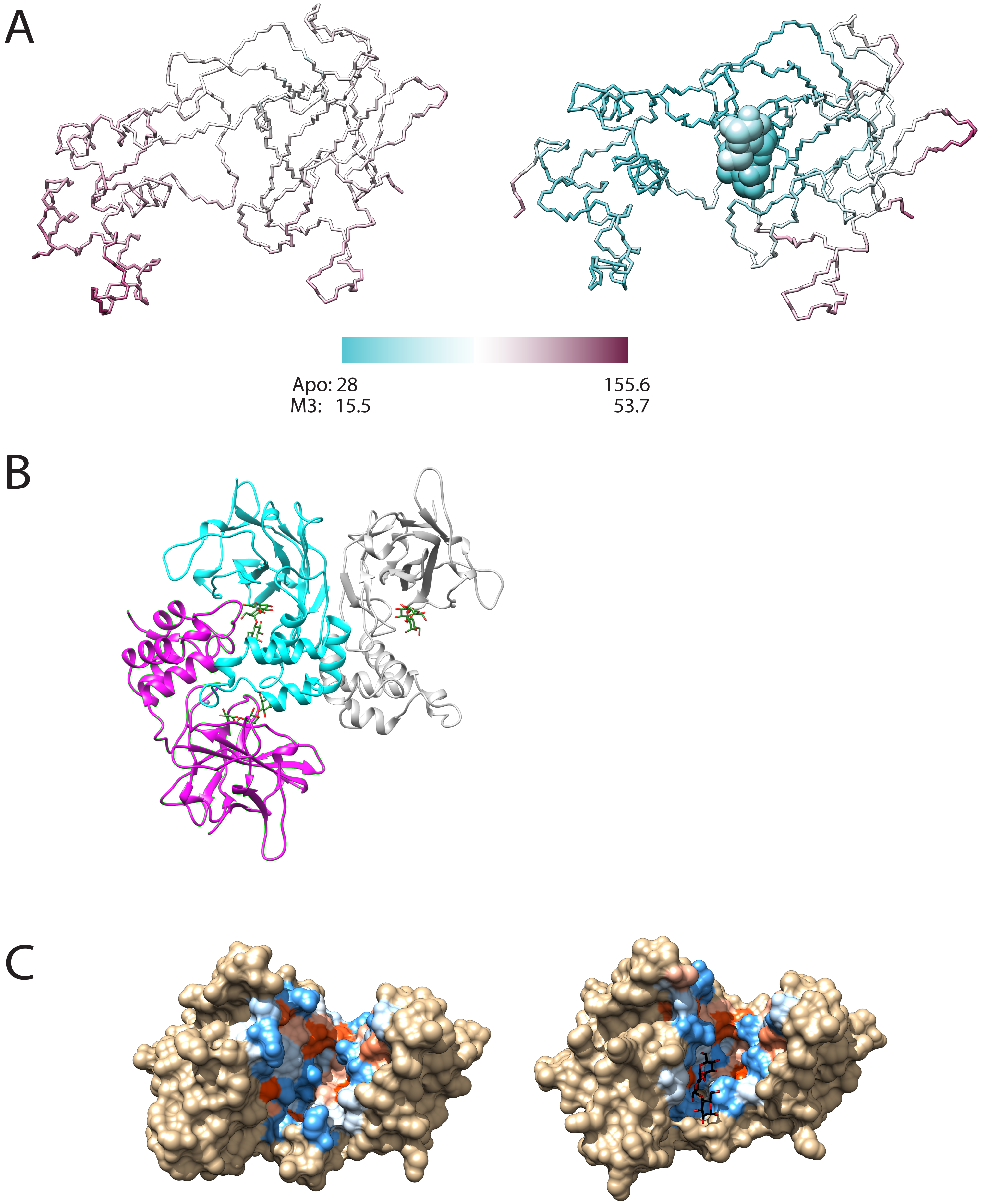

Supplemental Figure 2

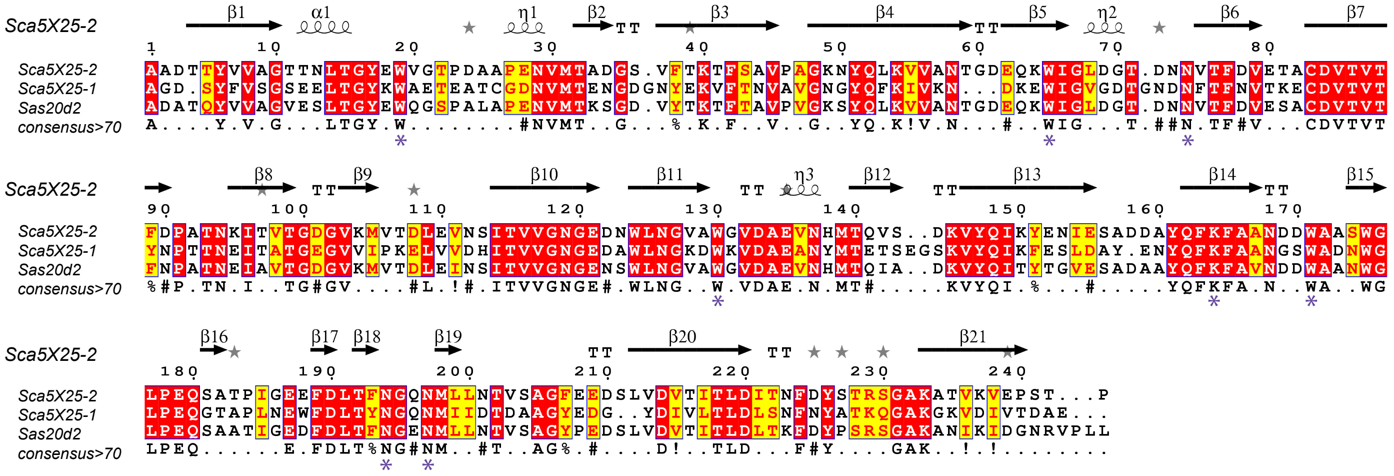

Supplemental Figure 3

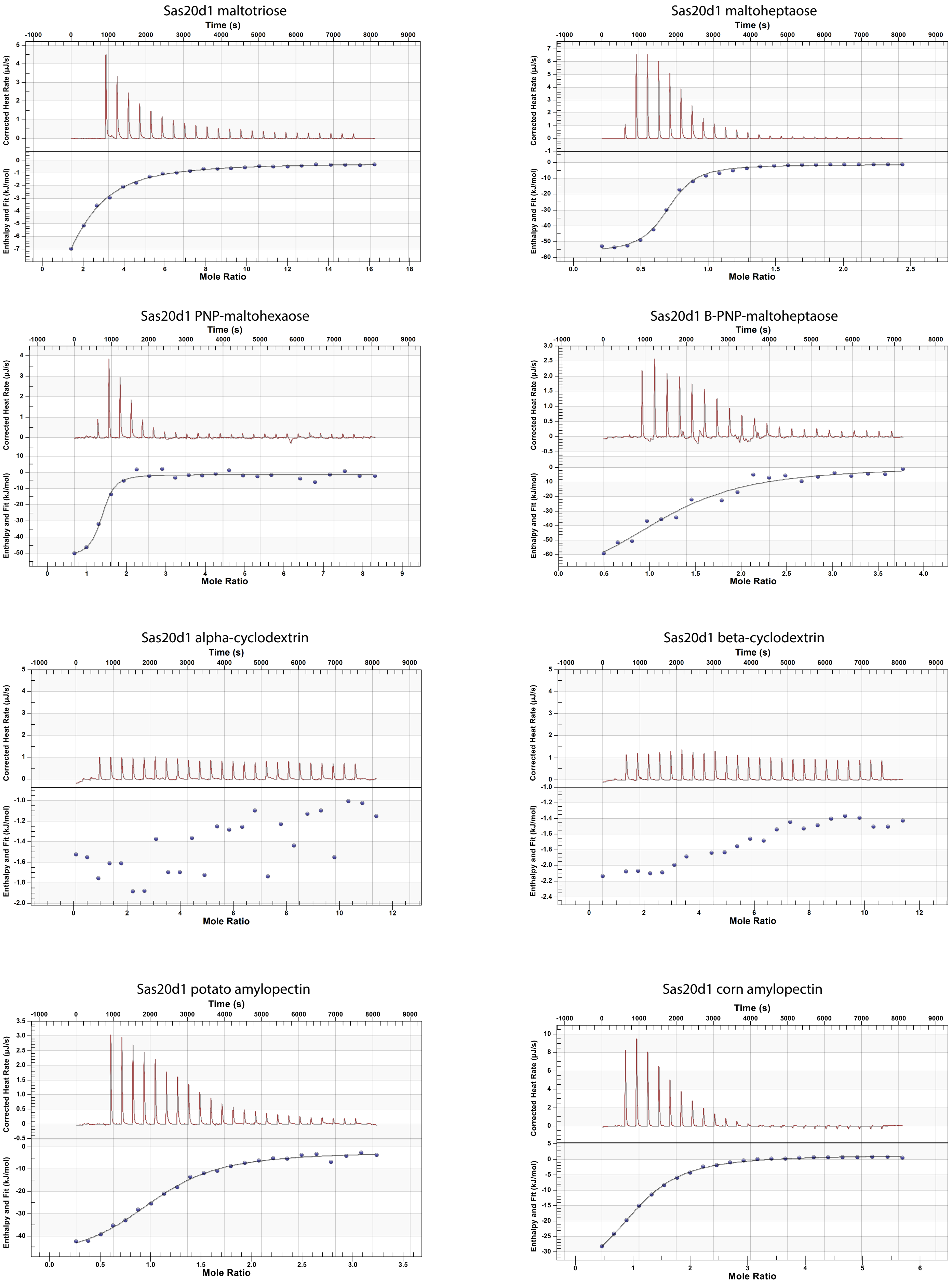

Supplemental Figure 4

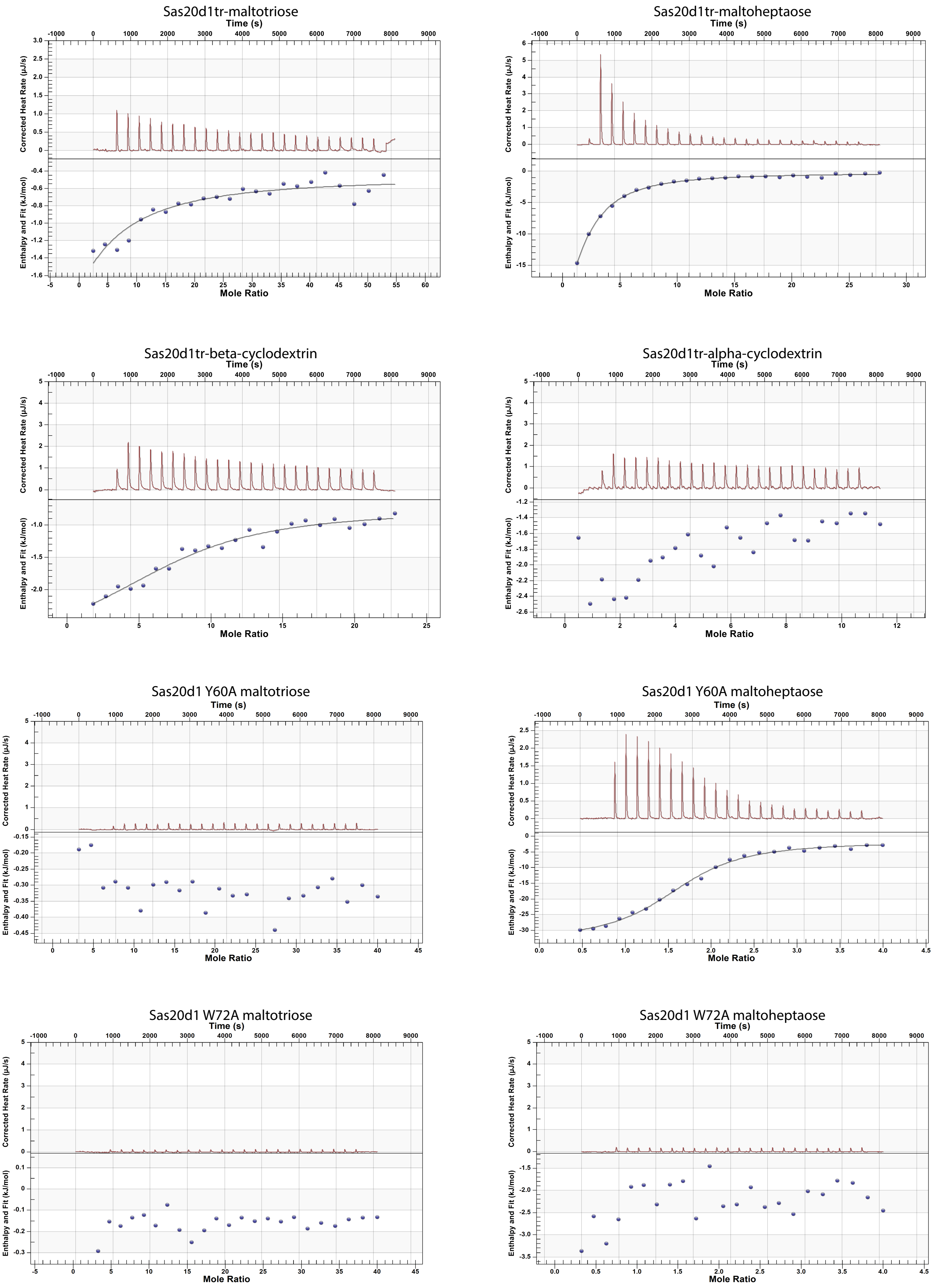

Supplemental Figure 5

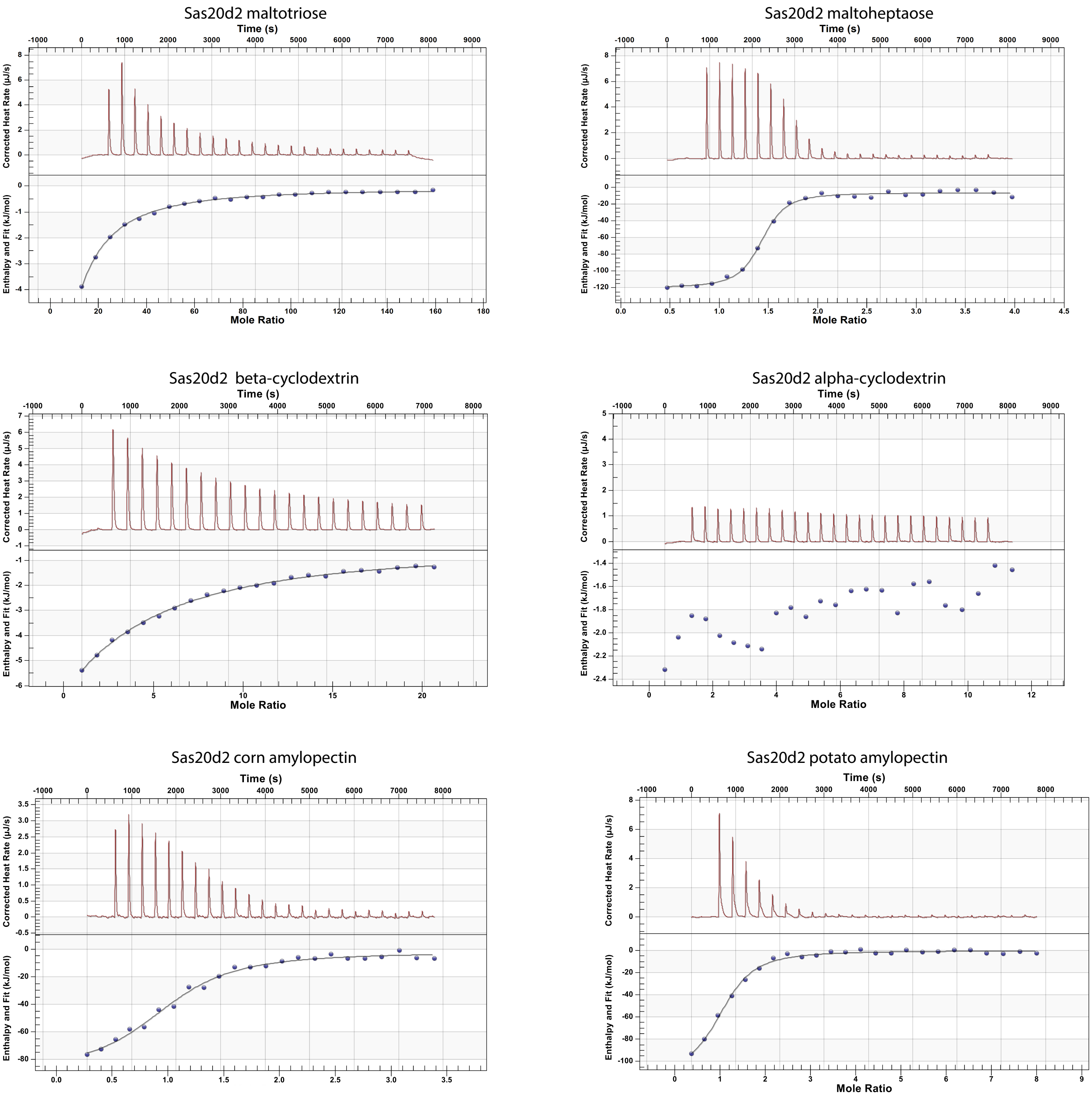

Supplemental Figure 6

Sas20d2 W329A maltotriose

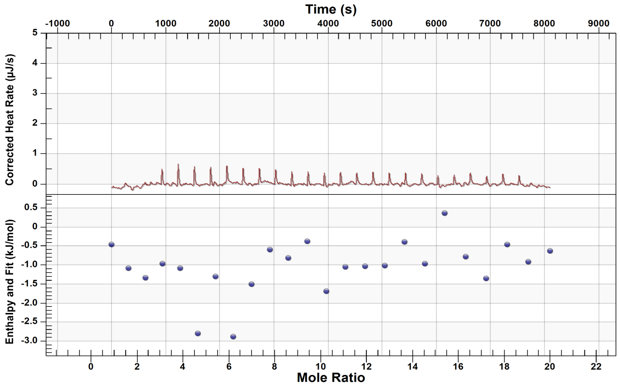

Sas20d2 W329A maltoheptaose

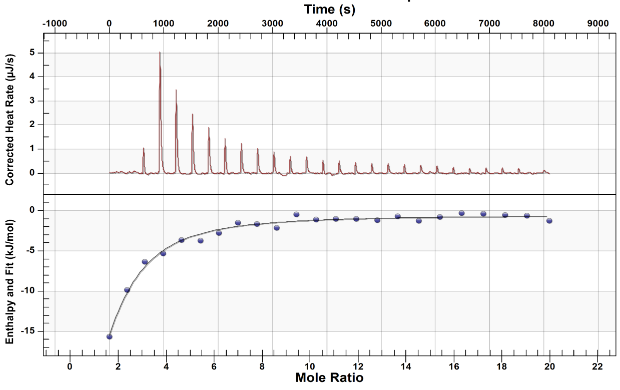

Sas20d2 W375A maltotriose

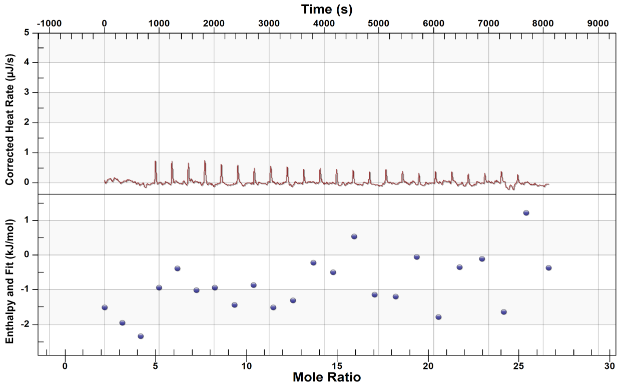

Sas20d2 W375A maltoheptaose

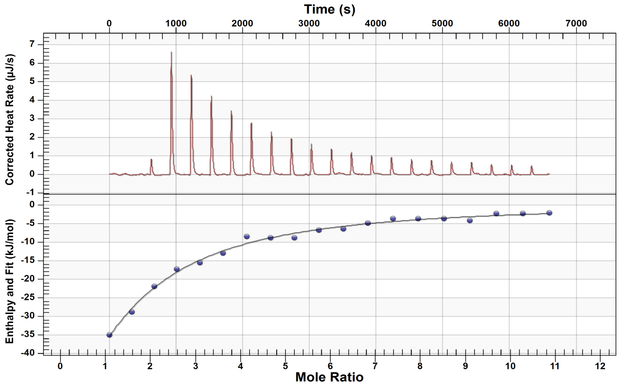

Sas20d2 W440A maltotriose

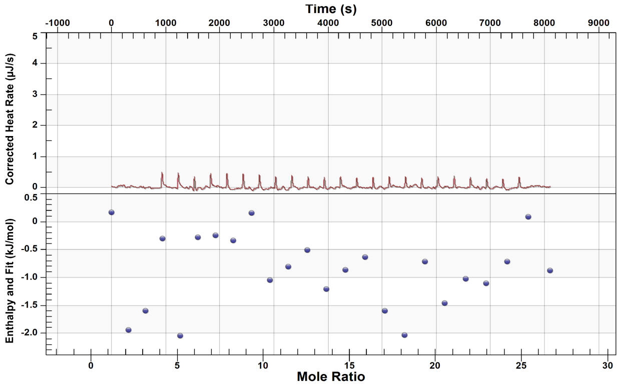

Sas20d2 W440A maltoheptaose

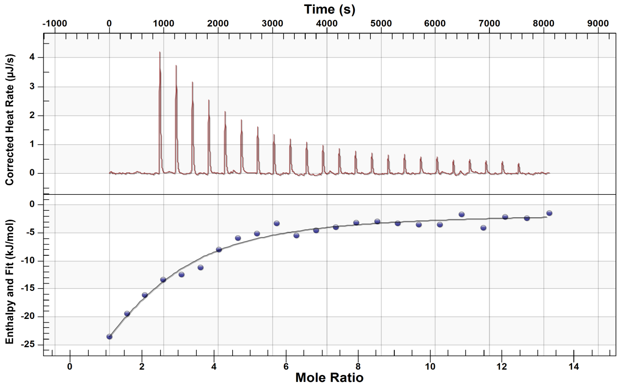

Sas20d2 W481A maltotriose

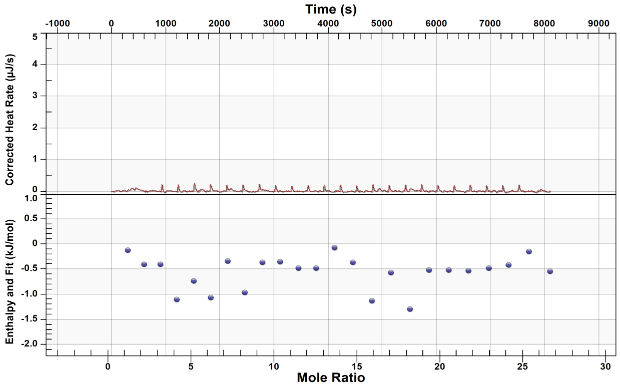

Sas20d2 W481A maltoheptaose

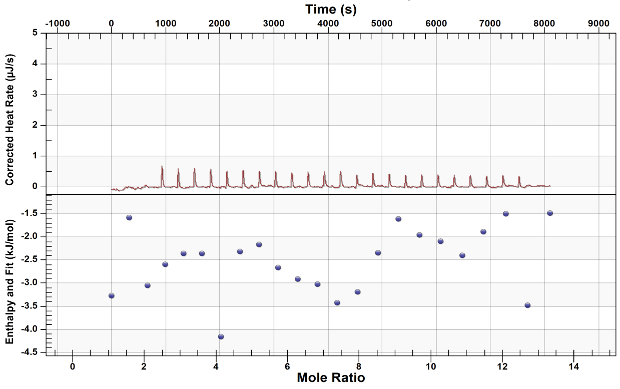

Supplemental Figure 7

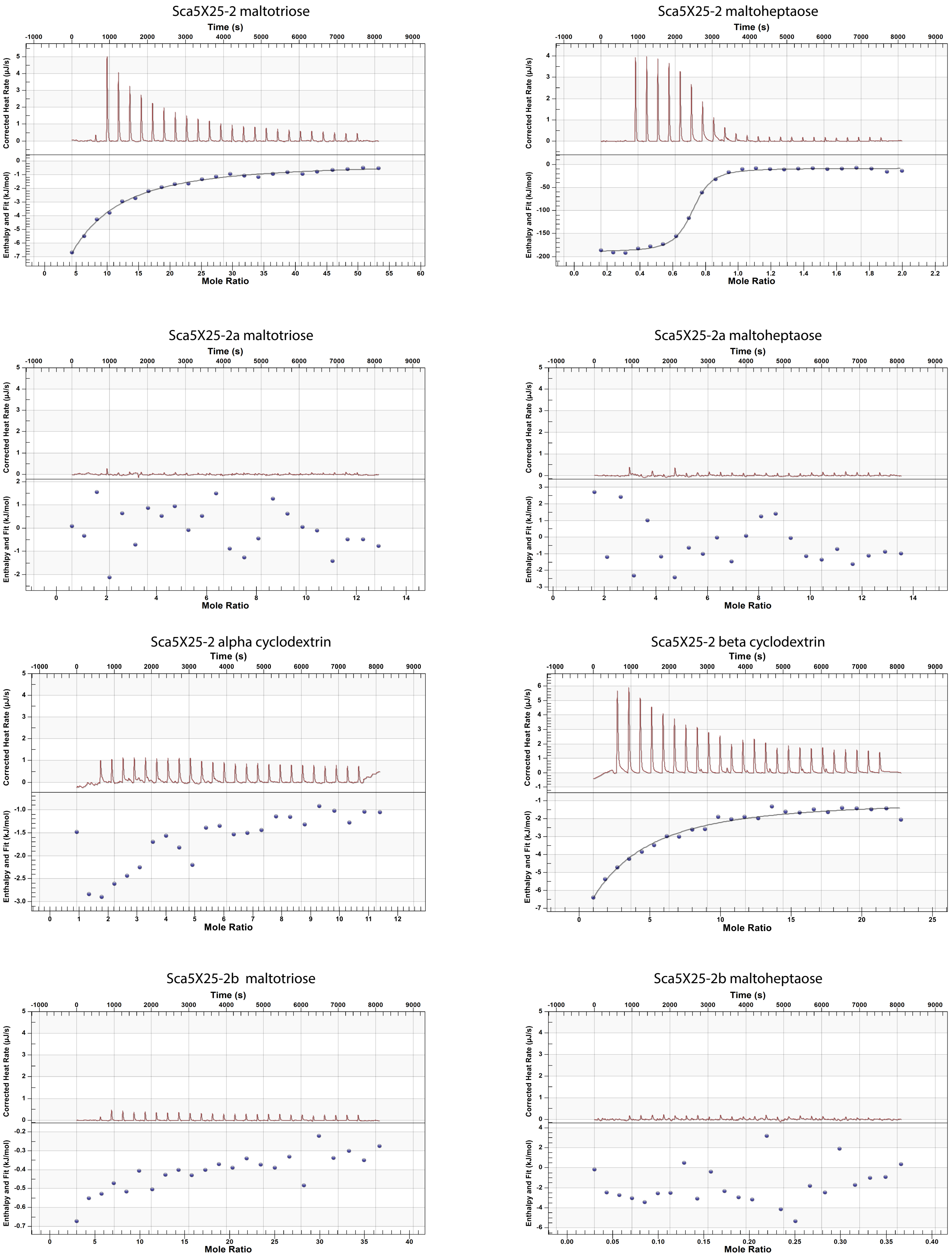

Supplemental Figure 8

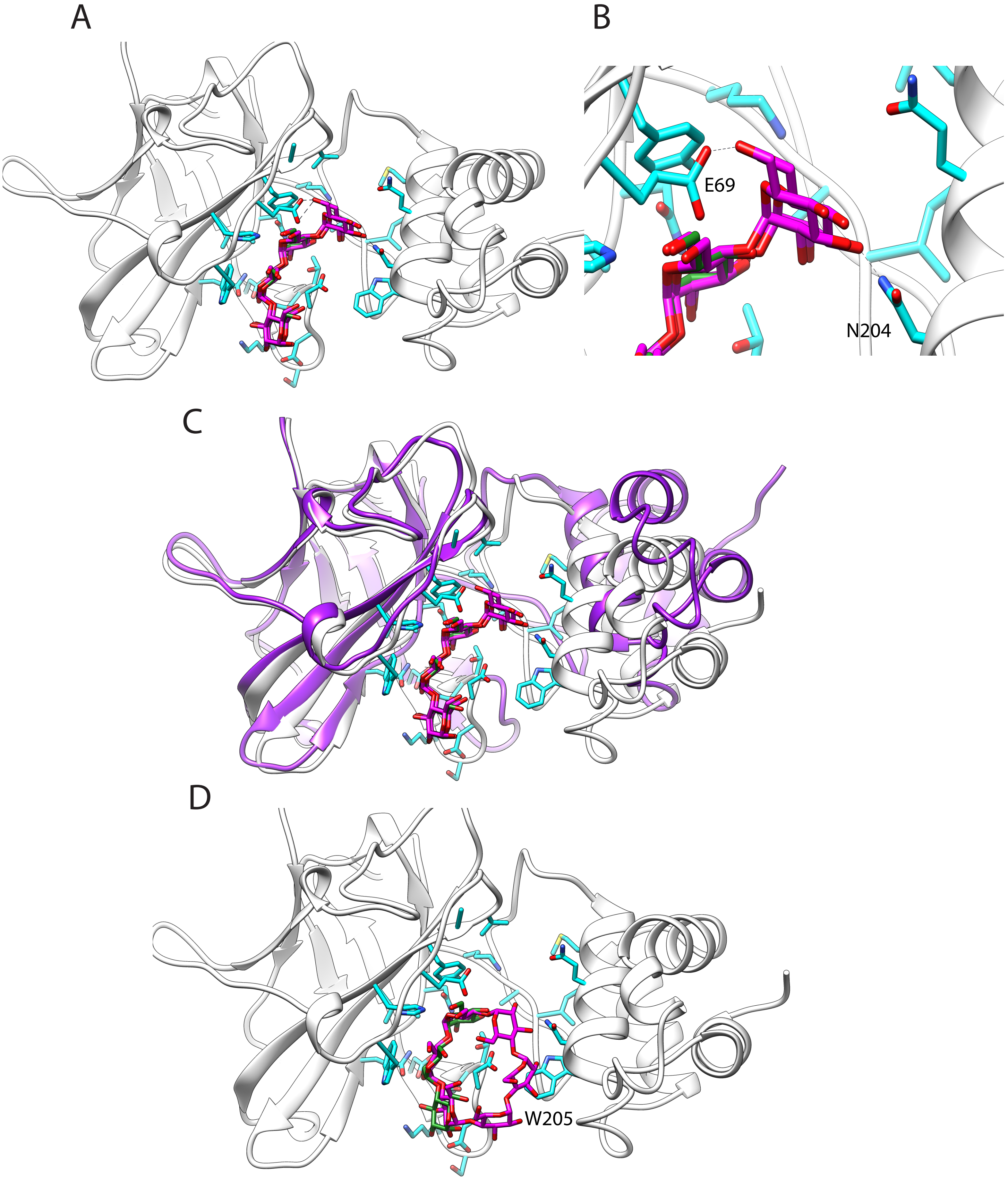

Supplemental Figure 9

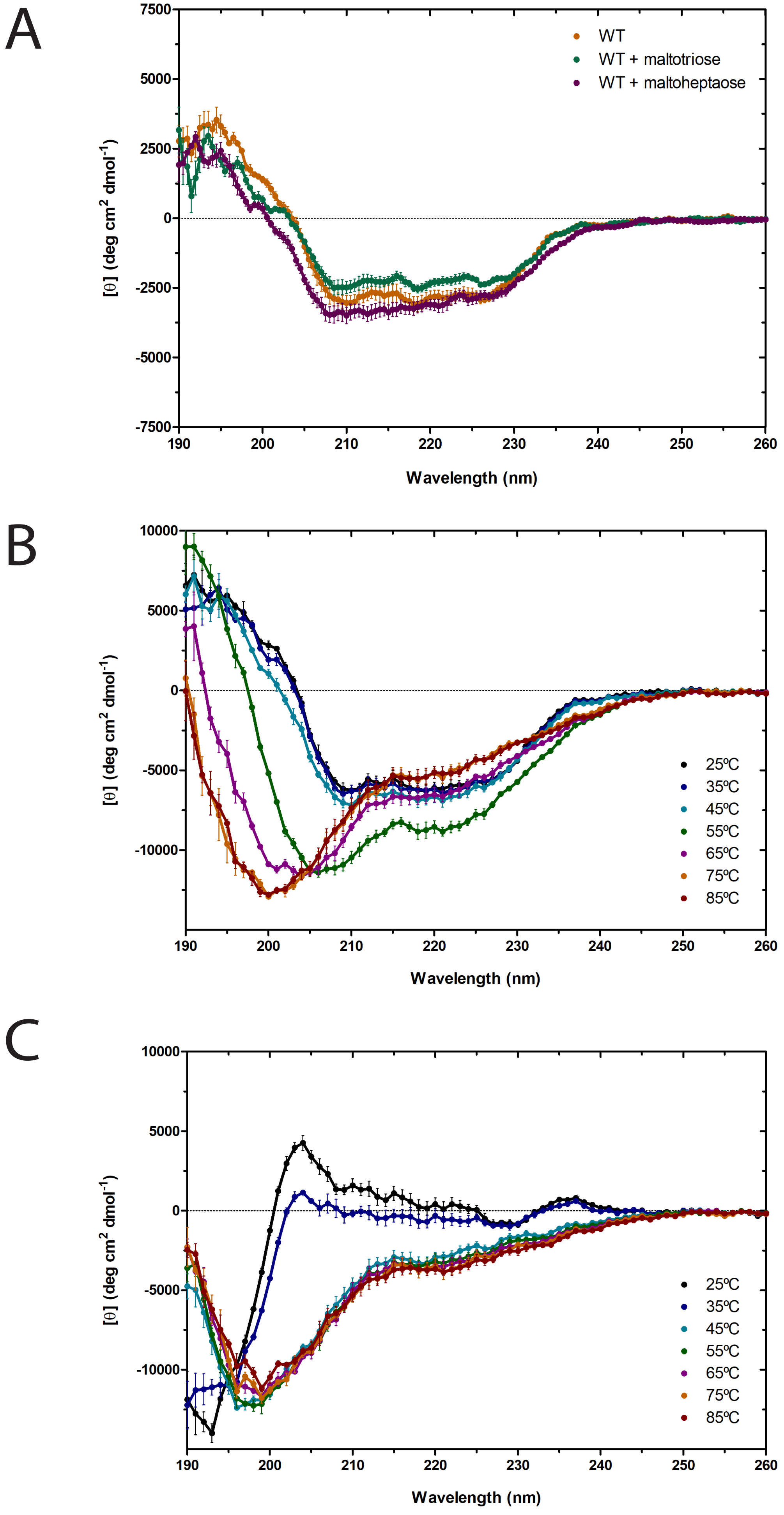

Supplementa Figure 10

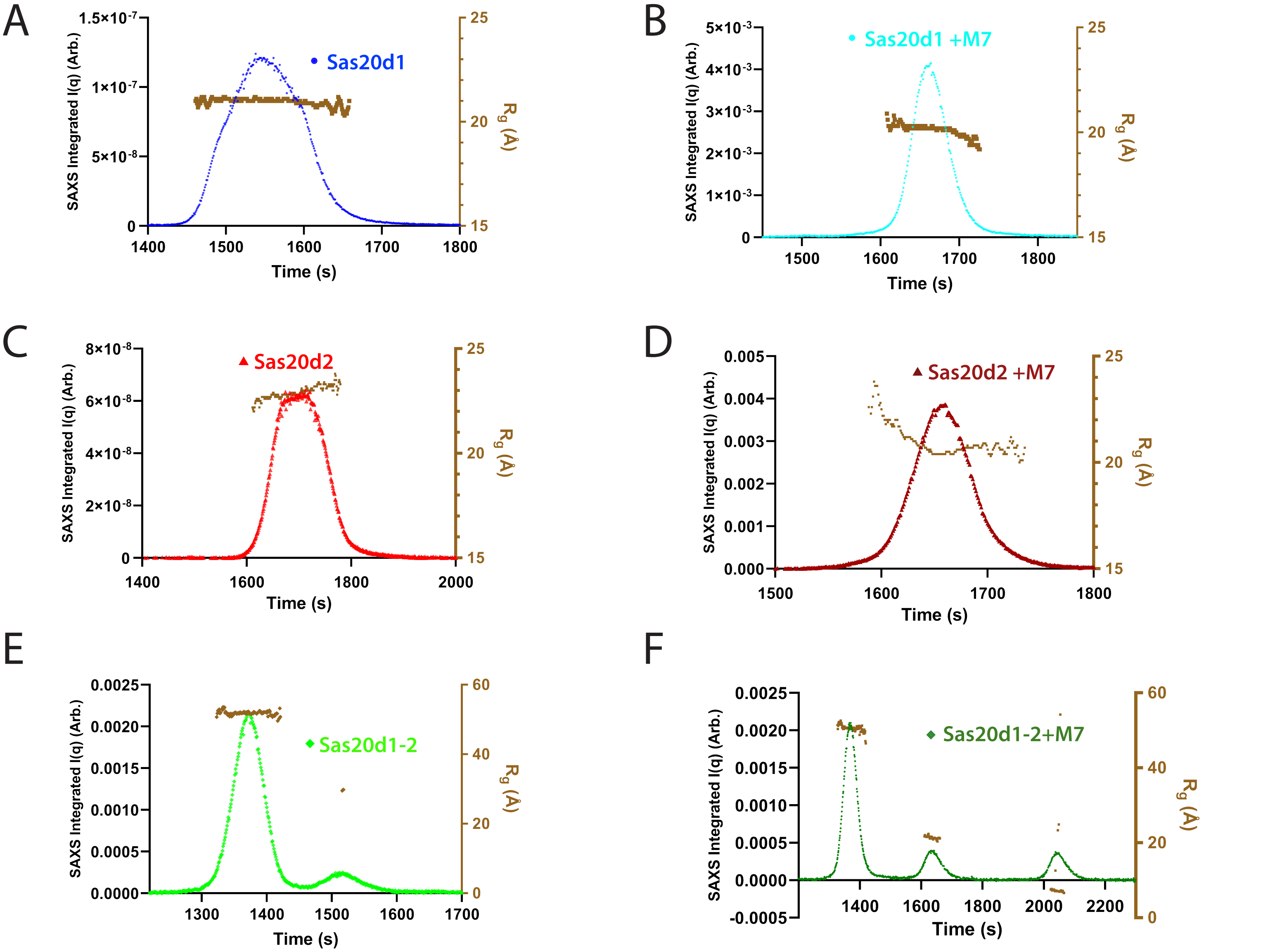

Supplemental Figure 11

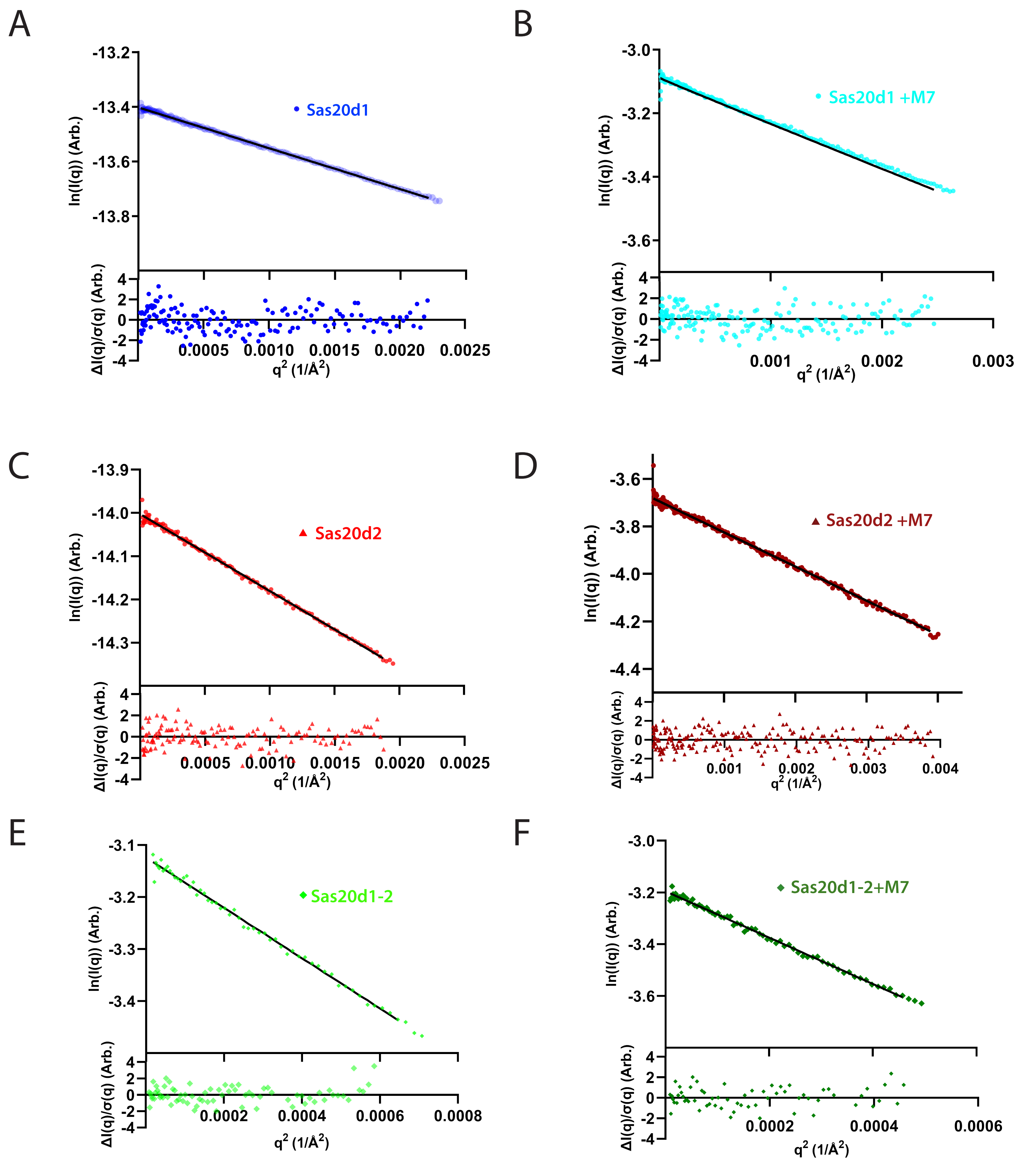

Supplemental Figure 12

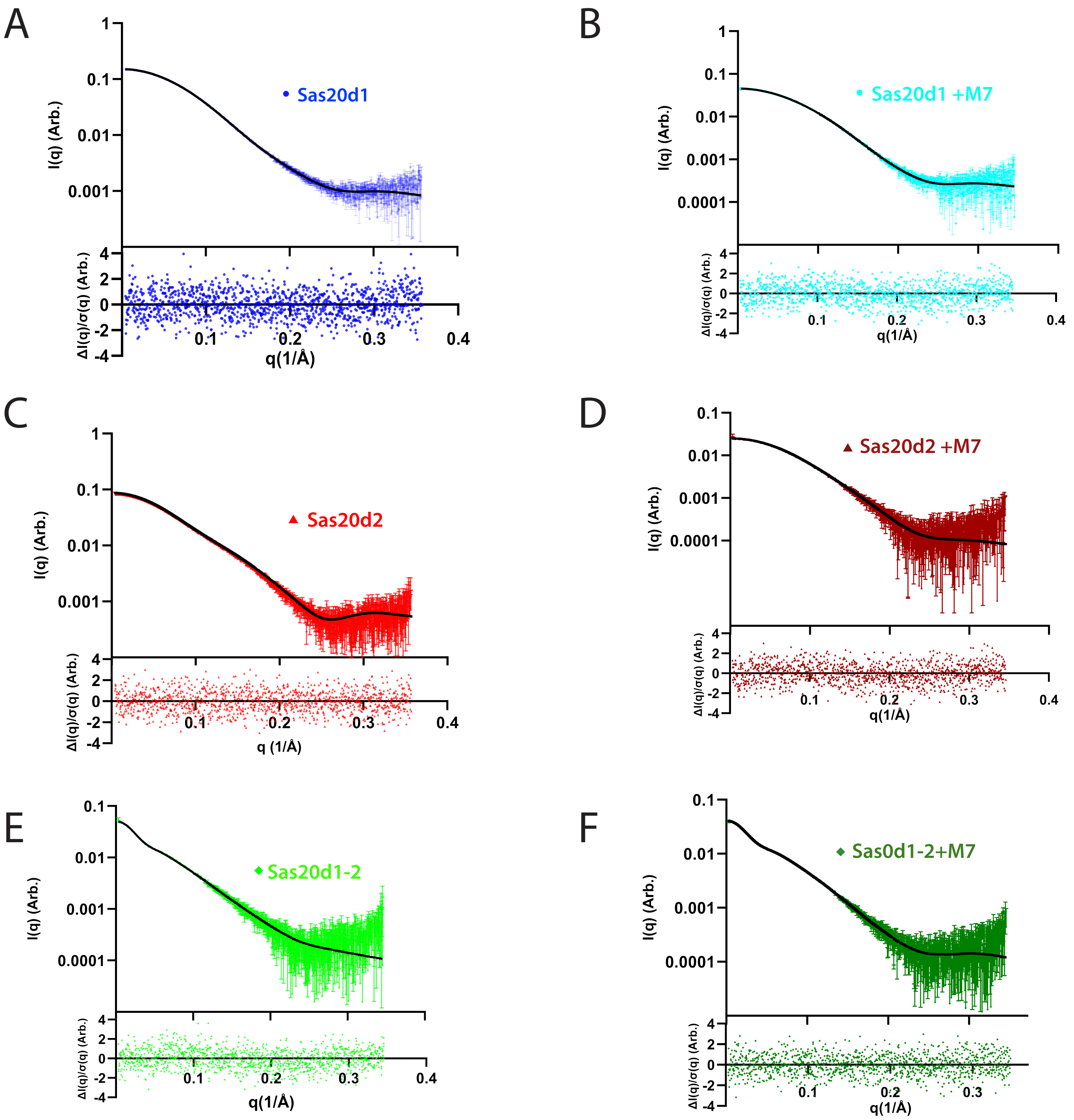

Supplemental Figure 13

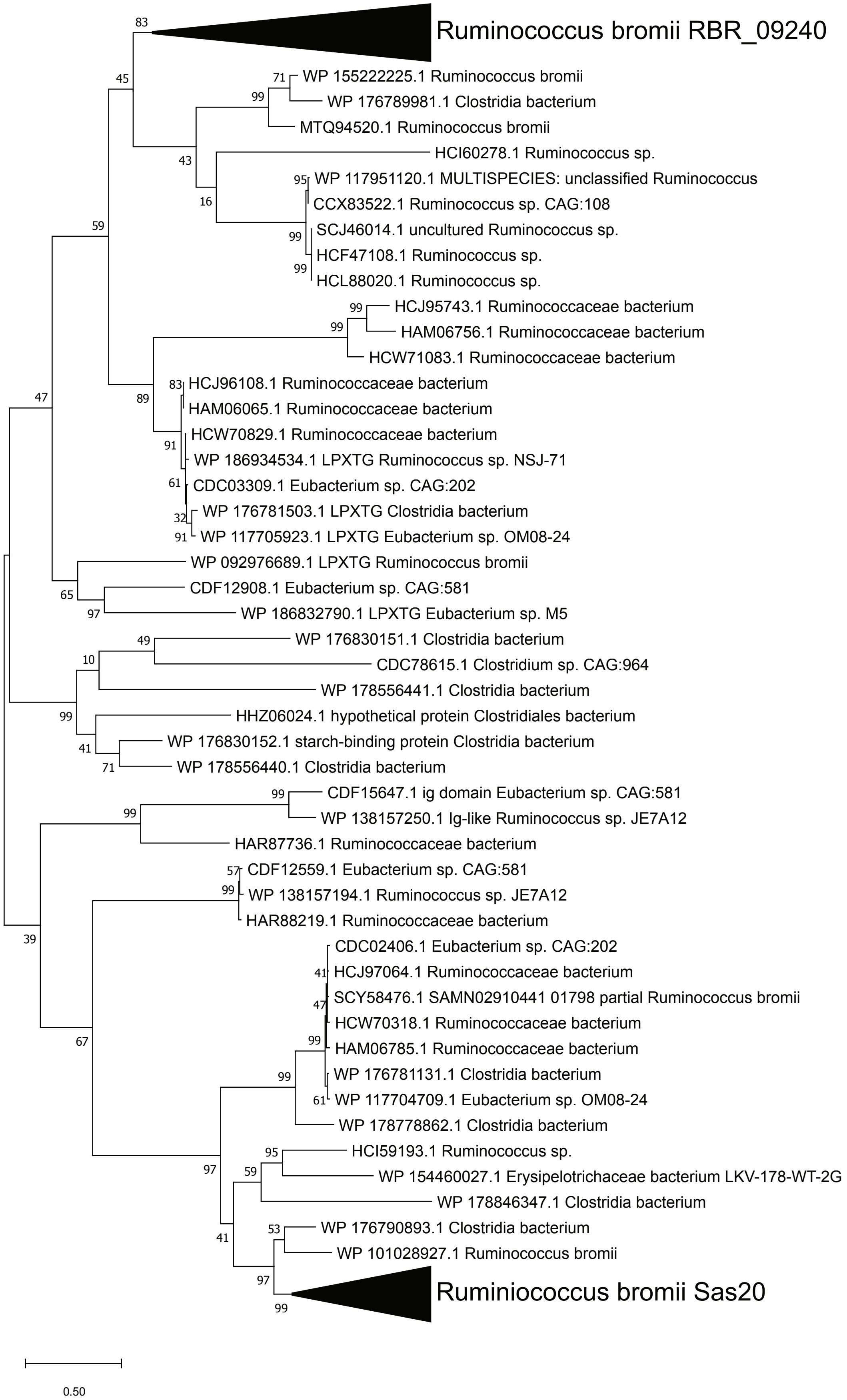

Supplemental Figure 14

```
                                                 * *           *
Sas20_Domain1    37 -------KIYFDASNLPAEWGTTKTVYCHLYAVAGD-DLPETSWQGKAEKCKKDTATGLY
RBR_09240        57 SEVEAGNVVKFDVK--KSGWNNVKSVFCHIWKADGSGDW--PAWQSKKEKCKYDSSTGIA

                                                          *  *
Sas20_Domain1    89 YFDTAKLKSADGTNHGGLKDNADYAVIFSTIDTKSQSHQTCNVTLGKPCLGDTIYLTGGT
RBR_09240       113 TYDLSKTGNTI-----SKSDGRVYCVIFSA----NTGMQSYNAIMSGKCIGDTLYCTGNQ

                       * *  *
Sas20_Domain1   149 VENTEDSSKRDFAATWKNNSDNYGPKAAITSLGHVTEGRFPIYLSRAEMVAQAIFNWAVK
RBR_09240       164 LENPEDSEKKANEAKWENNSD-CGPEKKITSTGNIIGSAFPEGESDATLLATYLVAYYND

Sas20_Domain1   209 NPKNYTPETVADICAQVEAEPMDVYNAYAE-MYATELADPA-------------------
RBR_09240       223 EAKTSFTQ---KILDEIKVSPTQVMGAVTDRINATKNPDKDTIAPAVEKILAGCTDPTTG

Sas20_Domain1   249 -----AYPDCAPLTTVATLLGVDPSGTTAPATEEPTT-----------------------
RBR_09240       280 KKVDKTQLDNAKKTGAKAAAGSSSNGSSSSSSNGGSSSSGSGSSSTGAVKSGVETTIVFV

Sas20_Domain1       ---------------------
RBR_09240       340 MAGLMVSAAGVMFLARKKKED
```

Supplemental Figure 15

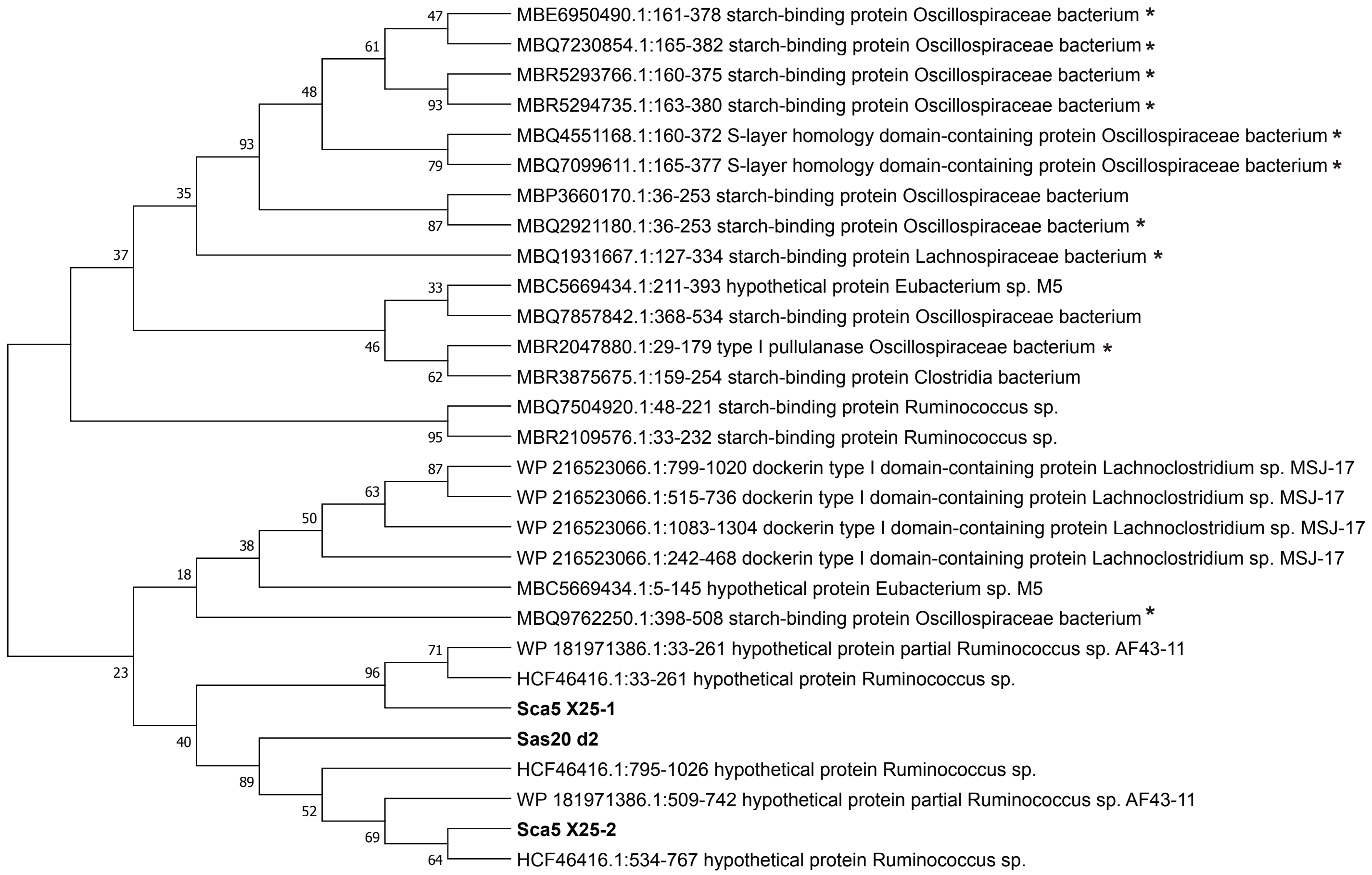

Supplemental Figure 16

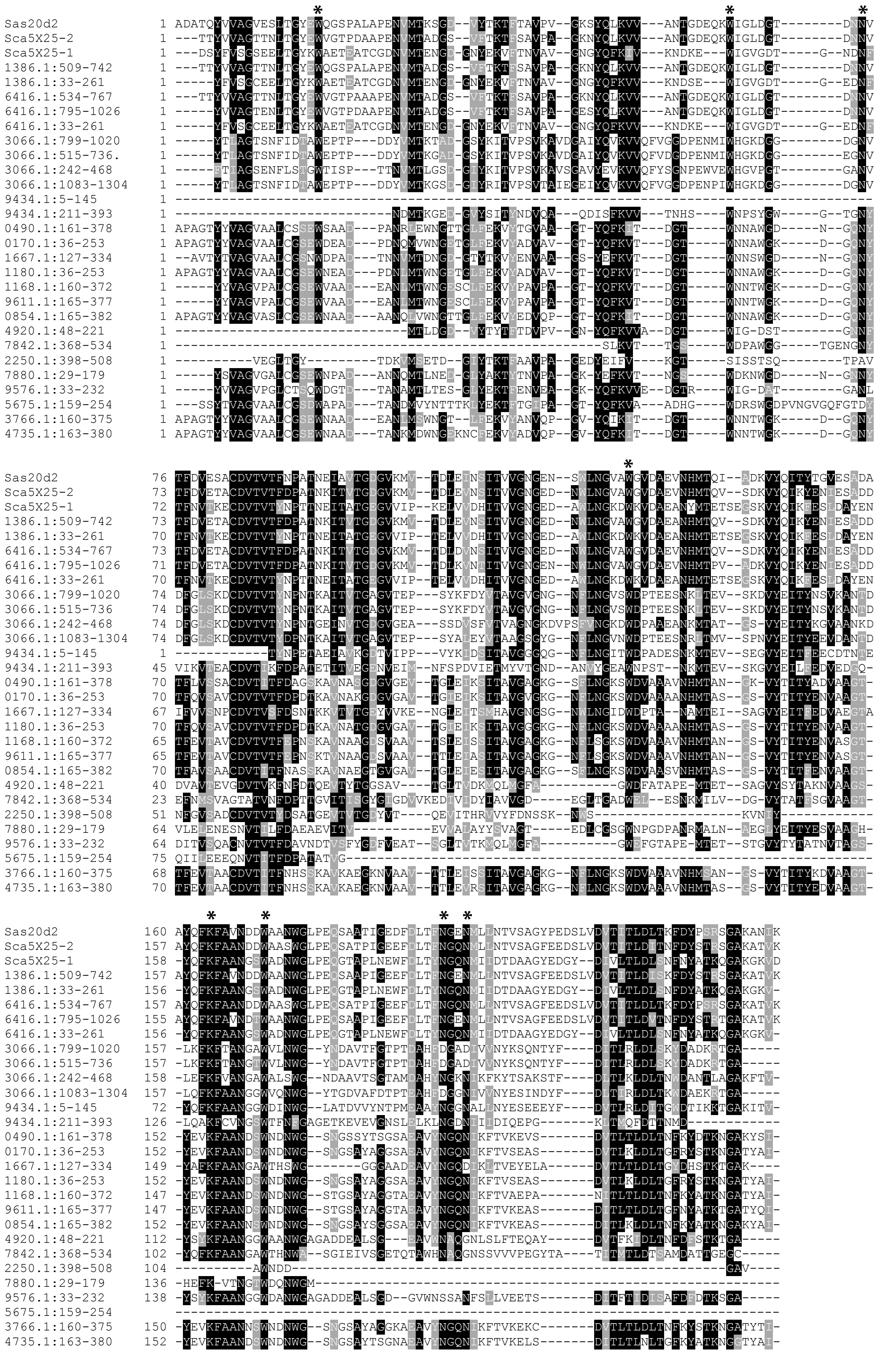

Supplemental Figure 17

```
Sas20d2a      1 ADATQYVVAGVES---LTGYEWQGSPALAPENVMTKSG--D-VYTKTFTAVPV-GKSYQL
Sas20d2b      1 LEINSITVVGNGENSWLNGVAWGVD---AEVNHMTQIA--DKVYQITYTGVESADAAYQF
Sca5X251a     1 AAGDSYFVSGSEE---LTGYKWAETEATCGDNVMTENG--DGNYEKVFTNVAV-GNGYQF
Sca5X251b     1 LVVDHITVVGNGEDAWLNGKDWKVD---AEANYMTETSEGSKVYQIKFESLDA-YENYQF
Sca5X252a     1 D--TTYVVAGTTN---LTGYEWVGTPDAAPENVMTADG--S-VFTKTFSAVPA-GKNYQL
Sca5X252b     1 LEVNSITVVGNGEDNWLNGVAWGVD---AEVNHMTQVS--DKVYQIKYENIESADDAYQF
Sca3X25a      1 SVTGDIALPL-------------------------AD-DGNGIYTGS---TELEAGSYTF
Sca3X25b      1 SIVGDINLDL-------------------------QATKDANVYSAS---VKLNKGNYEF
Sca3X25c      1 SVVGNFTLAL-------------------------AKTDKENVYSGT---KTLKAGNYQI
Sca3X25d      1 KVTGDINLSL-------------------------KK-SDTNVFTGS---VKLKKGDYSF

Sas20d2a     54 KVVANTGD-----------------EQKWIGLD------GTD-NNVTFD-----------
Sas20d2b     56 KFAVNDDW-----------------AANWGLPEQSAATIGE-DFDLTFNGENMLLNTVSA
Sca5X251a    55 KIVKND--------------------KEWIGVG------DTGNDNFTFN-----------
Sca5X251b    57 KFAANGSW-----------------ADNWGLPEQGTAPLNE-WFDLTYNGQNMIIDTDAA
Sca5X252a    52 KVVANTGD-----------------EQKWIGLD------GTD-NNVTFD-----------
Sca5X252b    56 KFAANDDW-----------------AASWGLPEQSATPIGE-EFDLTFNGQNMLLNTVSA
Sca3X25a     32 KMSVNGVAFGNGSTFTDKTTN-AKYNSKWTSS--------------TTL-----------
Sca3X25b     33 RVMNQGVRYCCGYTYKDLTVG-SQYNSKWSSA--------------STL-----------
Sca3X25c     33 KMNNYGKLCGTSAVVKDVTPG-LVFNPKWSKY--------------TTF-----------
Sca3X25d     32 KMNVNGTDFCNGTTIRNATTGTIKYNSKYTSA--------------STL-----------

Sas20d2a     79 ---VESACDVTVTFNPATNEIAVTGDGVKMVTD
Sas20d2b     98 GYPEDSLVDVTITLDLTKFD-YPSRSGAKANIK
Sca5X251a    78 ---VTKECDVTVTYNPTTNEITATGEGVVIPKE
Sca5X251b    99 GY--EDGYDIVLTLDLSNFN-YATKQGAKGKVD
Sca5X252a    77 ---VETACDVTVTFDPATNKITVTGDGVKMVTD
Sca5X252b    98 GFEEDSLVDVTITLDITNFD-YSTRSGAKATVK
Sca3X25a     66 ---KATGGKYTFKFNTAKNTLTIEY---------
Sca3X25b     67 ---AASGGTYTFSYDIDTNKLTISY---------
Sca3X25c     67 ---VATGGTYTFTYNTATNTLVVTA---------
Sca3X25d     67 ---VAVGGTYTFAYNALTNQLSVKYS-------K
```

Supplemental Figure 18
