# Supplementary material for: Sas20 is a highly flexible starch-binding protein in the Ruminococcus bromii cell-surface amylosome
Source: J Biol Chem. 2022 Apr 1;298(5):101896. doi: 10.1016/j.jbc.2022.101896 (PMC9112005; doi:10.1016/j.jbc.2022.101896)
Supplement: Supplemental Tables S2–S5 [file mmc3.docx]

**Table S2:** **Calculated secondary structure of Sas20d1 via circular dichroism.**

|  | Secondary Structure (%) | | | |
| --- | --- | --- | --- | --- |
| Condition | α-Helix | β-Strand | β-Turn | Unordered |
| Sas20d1 | 11.7 ± 1.5 | 33.3 ± 1.5 | 23.3 ± 0.6 | 31.7 ± 0.6 |
| Sas20d1, M3 | 9.7 ± 0.6 | 34.7 ± 0.6 | 23.3 ± 0.6 | 31.3 ± 1.0 |
| Sas20d1, M7 | 13.0 ± 1.7 | 30.0 ± 1.7 | 25.0 ± 0.0 | 32.0 ± 0.0 |

Values represent the means ± SD based on three replications. M3= maltotriose, M7= maltoheptaose. Traces displayed in Figure S10A.

**Table S3: Calculated secondary structure of apo Sas20d1 and Sas20d1tr via thermal circular dichroism.**

|  | Secondary Structure (%) | | | |
| --- | --- | --- | --- | --- |
| Condition | α-Helix | β-Strand | β-Turn | Unordered |
| Sas20d1 25ºC | 11.0 ± 1.0 | 33.3 ± 0.6 | 23.0 ± 0.0 | 32.0 ± 1.0 |
| Sas20d1 55ºC | 19.7 ± 1.2 | 22.33 ± 1.5 | 26.3 ± 1.5 | 31.7 ± 0.6 |
| Sas20d1 85ºC | 11.3 ± 1.5 | 28.7 ± 1.5 | 25.0 ± 0.0 | 34.7 ± 0.6 |
| Sas20d1tr 25ºC | 2.7 ± 0.6 | 39.3 ± 0.6 | 23.0 ± 1.0 | 32.3 ± 0.6 |
| Sas20d1tr 85ºC | 8.7 ± 2.1 | 31.7 ± 2.3 | 24.7 ± 0.6 | 34.7 ± 0.6 |

Values represent the means ± SD based on three replications. Traces are displayed in Figure S10B,C.

**Table S4A: Sample Parameters for SAXS Data Collection**

|  | Sas20d1 | Sas20d2 | Sas20d1-2 |
| --- | --- | --- | --- |
| Organism | *Ruminococcus bromii* L2-63 |  |  |
| Source | *E. coli* Rosetta (DE3) pLyS |  |  |
| UnitProt sequence ID (residues in construct) | R5DX05 (31-270) | R5DX05 (311-577) | R5DX05 (31-577) |
| Extinction coefficient [A_280_, 0.1%(w/v)] | 1.674 | 1.757 | 1.561 |
| \| Partial specific volume from  chemical composition (cm^3^ g^−1^) \| \| --- \| | 0.726 | 0.73 | 0.727 |
| \| Particle contrast from sequence and solvent constituents, (ρprotein − ρsolvent; 10^10^ cm^−2^) \| \| --- \| | 6.453 (9.457-3.004) | 6.523 (9.457-2.34) | 7.051 (9.525-2.915) |
| Mass from chemical composition (Da) | 26,000 | 26,400 | 56,000 |
| Superdex 200 10/300 Increase |  |  |  |
| Loading concentration (mg/mL) | 36 | 17 | 36 |
| Injection volume (μL) | 150 | 200 | 200 |
| Flow rate (mL/min) | 0.6 | 0.6 | 0.6 |
| Solvent | 1X PBS, 1mM TCEP, pH=7 |  |  |

**Table S4B: Instrumentation and Data Collection Protocols for SAXS**

| \| Instrument \| BioCAT facility at the Advanced Photon Source beamline 18ID  with Pilatus3 X IM (Dectris) detector \| \| --- \| --- \| \| Wavelength (Å) \| 1.033 \| \| Beam size (μm^2^) \| 150 (h) x 25 (v) focused at the detector \| \| Camera length (m) \| 3.629 \| \| q-measurement range (Å-1) \| .0042-.36 \| \| Absolute scaling method \| Glassy Carbon, NIST SRM 3600 \| \| Basis for normalization to constant counts \| To transmitted intensity by beam-stop counter \| \| Method for monitoring radiation damage \| Automated frame-by-frame comparison of relevant regions using CORMAP implemented in BioXTAS RAW \| \| Exposure time, number of exposures \| 0.5 s exposure time with a 1 s total exposure period (0.5 s on, 0.5 s off) of entire SEC elution \| \| Sample configuration \| SEC-SAXS with sheath -flow cell, effective path length 0.542 mm. Size separation by an AKTA Pure with a Superdex 200 10/300 Increase column \| \| Sample temperature (ºC) \| 23 \| |
| --- | --- | --- | --- | --- | --- | --- | --- | --- | --- | --- | --- | --- | --- | --- | --- | --- | --- | --- | --- | --- | --- | --- |

**Table S4C: Software employed for SAS data reduction, analysis, and interpretation**

| SAXS data reduction | Radial averaging; frame comparison, averaging, and subtraction done using BioXTAS RAW 2.1.0 |
| --- | --- |
| Basic analysis: Guinier, M.W., P(r) | Guinier fit and M.W. using BioXTAS RAW, P(r) function using GNOM (Svergun, 1992). RAW uses MoW and Vc M.W. methods (Rambo & Tainer, 2013; Piiadov et al., 2018) |

**Table S5: Primer Table**

| Name | Sequence | Purpose |
| --- | --- | --- |
| Sas20d1f | **CATCATCACCACCATCACGAGAACCTGTA**  **CTTCCAGGGC** TTCTCTGCATCAGCTGAAGAAACC | Forward primer for Sas20d1 and Sas20d1-2 constructs |
| Sas20d1Ar | **GTGGCGGCCGCTCTATTA** TCTGCTCTTGAAAGGTAA | Reverse primer for Sas20d1A construct |
| Sas20d1r | **GTGGCGGCCGCTCTATTA** AGGAGCTGTTGTACCTGAAGG | Reverse primer for Sas20d1 construct |
| Sas20d2f | **CATCATCACCACCATCACGAGAACCTGTA CTTCCAGGGC** GAGCCTGCTGACGCAACACAG | Forward primer for Sas20d2 construct |
| Sas20d2r | **GTGGCGGCCGCTCTATTA** AGCATCGCCGAGAAGCGGAACACG | Reverse primer for Sas20d2 and Sas20d1-2 constructs |
| Sca5X25-2f | **CATCATCACCACCATCACGAGAACCTGTA CTTCCAGGGC** GCTGCCGATACTACATATGTA | Forward primer for Sca5X25-2 and Sca5X25-2a constructs |
| Sca5X25-2ar | **GTGGCGGCCGCTCTATTA** GTTAACTTCAAGGTCTGTTAC | Reverse primer for Sca5X25-2a |
| Sca5X25-2bf | **CATCATCACCACCATCACGAGAACCTGTA CTTCCAGGGC** ACAGACCTTGAAGTTAAC | Forward primer for Sca5X25b |
| Sca5X25-2r | **GTGGCGGCCGCTCTATTA** AGTTGAAGGCTCAACCTTAAC | Reverse primer for Sca5X25-2 and Sca5X25-2b |
| Sas20d1Y60Af | GTATATTGTCACCTTgccGCTGTAGCTGGCGAT | Forward primer for Sas20d1 Y60A mutant |
| Sas20d1Y60Ar | ATCGCCAGCTACAGCggcAAGGTGACAATATAC | Reverse primer for Sas20d1 Y60A mutant |
| Sas20d1W72Af | TTACCTGAAACATCTgcgCAGGGTAAGGCAGAG | Forward primer for Sas20d1 W72A mutant |
| Sas20d1W72Ar | CTCTGCCTTACCCTGcgcAGATGTTTCAGGTAA | Reverse primer for Sas20d1 W72A mutant |
| Sas20d2W329Af | CTCACAGGTTATGAAgcgCAGGGTTCTCCTGCA | Forward primer for Sas20d2 W329A mutant |
| Sas20d2W329Ar | TGCAGGAGAACCCTGcgcTTCATAACCTGTGAG | Reverse primer for Sas20d2 W329A mutant |
| Sas20d2W375Af | GGCGACGAGCAGAAGgcgATCGGTCTTGACGGT | Forward primer for Sas20d2 W375A mutant |
| Sas20d2W375Ar | ACCGTCAAGACCGATcgcCTTCTGCTCGTCGCC | Reverse primer for Sas20d2 W375A mutant |
| Sas20d2W440Af | CTTAACGGTGTAGCAgcgGGCGTTGACGCTGAA | Forward primer for Sas20d2 W440A mutant |
| Sas20d2W440Ar | TTCAGCGTCAACGCCcgcTGCTACACCGTTAAG | Reverse primer for Sas20d2 W440A mutant |
| Sas20d2W481Af | GCAGTTAACGACGATgcgGCTGCTAACTGGGGT | Forward primer for Sas20d2 W481A mutant |
| Sas20d2W481Ar | ACCCCAGTTAGCAGCcgcATCGTCGTTAACTGC | Reverse primer for Sas20d2 W481A mutant |

Bold text denotes regions of homology for cloning into pETite vectors and engineered TEV cleavage site, lower case text denotes mutagenized region.
